# UCHL1 provides diagnostic and antimetastatic strategies due to its deubiquitinating effect on HIF-1α

**DOI:** 10.1038/ncomms7153

**Published:** 2015-01-23

**Authors:** Yoko Goto, Lihua Zeng, Chan Joo Yeom, Yuxi Zhu, Akiyo Morinibu, Kazumi Shinomiya, Minoru Kobayashi, Kiichi Hirota, Satoshi Itasaka, Michio Yoshimura, Keiji Tanimoto, Masae Torii, Terumasa Sowa, Toshi Menju, Makoto Sonobe, Hideaki Kakeya, Masakazu Toi, Hiroshi Date, Ester M. Hammond, Masahiro Hiraoka, Hiroshi Harada

**Affiliations:** 1 Department of Radiation Oncology and Image-applied Therapy, Kyoto University Graduate School of Medicine, 54 Shogoin Kawahara-cho, Kyoto 606-8507, Japan; 2 Group of Radiation and Tumor Biology, Career-Path Promotion Unit for Young Life Scientists, Kyoto University, Yoshida Konoe-cho, Kyoto 606-8501, Japan; 3 Department of Radiation Medicine, Fourth Military Medical University, 17 Changle West Road, Shaanxi 710032, China; 4 Department of Oncology, First Affiliated Hospital of Chongqing Medical University, No.1 Friendship Road, Yuanjiagang, Chongqing 400016, China; 5 Department of Anesthesia, Kyoto University Hospital, Kyoto University, 54 Shogoin Kawahara-cho, Kyoto 606-8507, Japan; 6 Department of Radiation Biology, Research Institute for Radiation Biology and Medicine, Hiroshima University, 1-2-3 Kasumi, Hiroshima 734-8553, Japan; 7 Department of Breast Surgery, Kyoto University Graduate School of Medicine, 54 Shogoin Kawahara-cho, Kyoto 606-8507, Japan; 8 Department of Thoracic Surgery, Kyoto University Graduate School of Medicine, 54 Shogoin Kawahara-cho, Kyoto 606-8507, Japan; 9 Division of Bioinformatics and Chemical Genomics, Graduate School of Pharmaceutical Sciences, Kyoto University, 46-29 Yoshida Shimoadachi-cho, Kyoto 606-8501, Japan; 10 CRUK/MRC Oxford Institute for Radiation Oncology, Department of Oncology, University of Oxford, Old Road Campus Research Building, Oxford OX3 7DQ, UK; 11 Precursory Research for Embryonic Science and Technology (PRESTO), Japan Science and Technology Agency (JST), 4-1-8 Honcho, Saitama 332-0012, Japan

## Abstract

Hypoxia-inducible factor 1 (HIF-1) plays a role in tumour metastases; however, the genes that activate HIF-1 and subsequently promote metastases have yet to be identified. Here we show that Ubiquitin C-terminal hydrolase-L1 (UCHL1) abrogates the von Hippel–Lindau-mediated ubiquitination of HIF-1α, the regulatory subunit of HIF-1, and consequently promotes metastasis. The aberrant overexpression of UCHL1 facilitates distant tumour metastases in a HIF-1-dependent manner in murine models of pulmonary metastasis. Meanwhile, blockade of the UCHL1–HIF-1 axis suppresses the formation of metastatic tumours. The expression levels of UCHL1 correlate with those of HIF-1α and are strongly associated with the poor prognosis of breast and lung cancer patients. These results indicate that UCHL1 promotes metastases as a deubiquitinating enzyme for HIF-1α, which justifies exploiting it as a prognostic marker and therapeutic target of cancers.

## Abstract (web summary)

When stabilized, HIF-1 can activate adaptation to hypoxia and metastasis. Here the authors show that upregulation of Ubiquitin C-terminal hydrolase-L1 in human cancers promotes metastasis and correlates with poor prognosis because of its role in opposing ubiquitin-mediated degradation of HIF-1.

## Graphical abstract

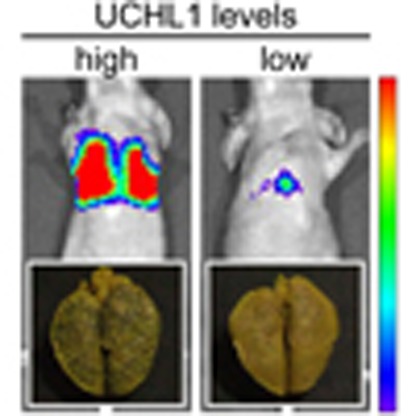

Metastasis is the major cause of death among cancer patients^1^. Previous studies have shown that hypoxia-inducible factor 1 (HIF-1) plays important roles in distant tumour metastases at multiple steps^2^,^3^. Clinical studies have also demonstrated that HIF-1 could be used as an adverse prognostic factor for both local tumour recurrence and distant tumour metastasis in cancer patients^4^,^5^. These findings have justified targeting HIF-1 for cancer therapies^6^,^7^.

HIF-1 is a heterodimer composed of an α-subunit (HIF-1α) and β-subunit (HIF-1β), and its activity is mainly regulated through oxygen-dependent changes in protein stability and the transactivation activity of HIF-1α^6^,^7^. Under normoxic conditions, prolyl-4-hydroxylases hydroxylate the proline residues, P_402_ and P_564_, in the oxygen-dependent degradation (ODD) domain of HIF-1α^8^,^9^. This hydroxylation triggers ubiquitination by a von Hippel–Lindau (VHL)-containing E3 ubiquitin ligase, which leads to the rapid proteolysis of HIF-1α^8^,^9^. In addition, the asparagynyl-hydroxylase, factor inhibiting HIF-1, hydroxylates the asparagine residue, N_803_, in the C-terminal transactivation domain of HIF-1α, resulting in the suppression of its transactivation activity^10^,^11^. In contrast, HIF-1α becomes stable and active under hypoxic conditions because of the inactivation of oxygen-dependent hydoxylases^8^,^9^. The resulting heterodimer, HIF-1, binds to its cognate enhancer sequence, the hypoxia response element (HRE), and induces the expression of various genes responsible for distant tumour metastases, angiogenesis and metabolic reprogramming^3^,^6^,^7^. Thus, the mechanism by which HIF-1 activity is influenced by oxygen availability has been characterized in detail; however, the genes that actively induce HIF-1 activity and consequently promote metastases remain largely unknown. Elucidating this molecular network, especially identifying the novel upstream activators of HIF-1, may provide a valuable insight that could eradicate secondary cancers because they can be exploited as targets for cancer therapy.

In the present study, we provided a novel insight into the regulatory mechanism underlying tumour metastasis via activation of the Ubiquitin C-terminal hydrolase-L1 (UCHL1)-HIF-1 pathway. We successfully identified UCHL1 as a novel upstream activator of HIF-1 through an original genetic screening strategy. The aberrant expression of UCHL1 was shown to promote distant tumour metastasis via the activation of HIF-1. Loss-of-function studies and an inhibitor for UCHL1 confirmed the importance of UCHL1 for cancer therapy. Moreover, expression levels of UCHL1 in breast and lung cancers were found to be associated with those of HIF-1α and poor prognosis in cancer patients.

## Results

### UCHL1 upregulates HIF-1 activity

We developed a new genetic screening method to explore the novel activators of HIF-1 with the expectation that they would include a prometastatic factor. Using an artificial promoter composed of five repeats of the HRE and the human cytomegalovirus (CMV) minimal promoter (5HRE promoter: herein designated as 5HREp)^12^,^13^, we constructed a p5HREp-bsd plasmid that expressed the blasticidin S-resistant gene (*bsd*) in a HIF-1-dependent manner (Supplementary Fig. 1a). The stable transfectant of NIH3T3 cells with p5HREp-bsd (NIH3T3/5HREp-bsd) was sensitive to blasticidin S under normoxic conditions because of its insufficient expression of both HIF-1α and BSD (Supplementary Fig. 1b). After introducing the human complementary DNA library, NIH3T3/5HREp-bsd cells were cultured in blasticidin S-containing medium under normoxic conditions as the resultant surviving colonies were expected to be introduced with cDNAs encoding an activator of HIF-1. We isolated several surviving colonies through this gene screening approach, and identified UCHL1 as a candidate for a novel upstream activator of HIF-1 by further analysing one of the surviving colonies.

**Figure 1:**
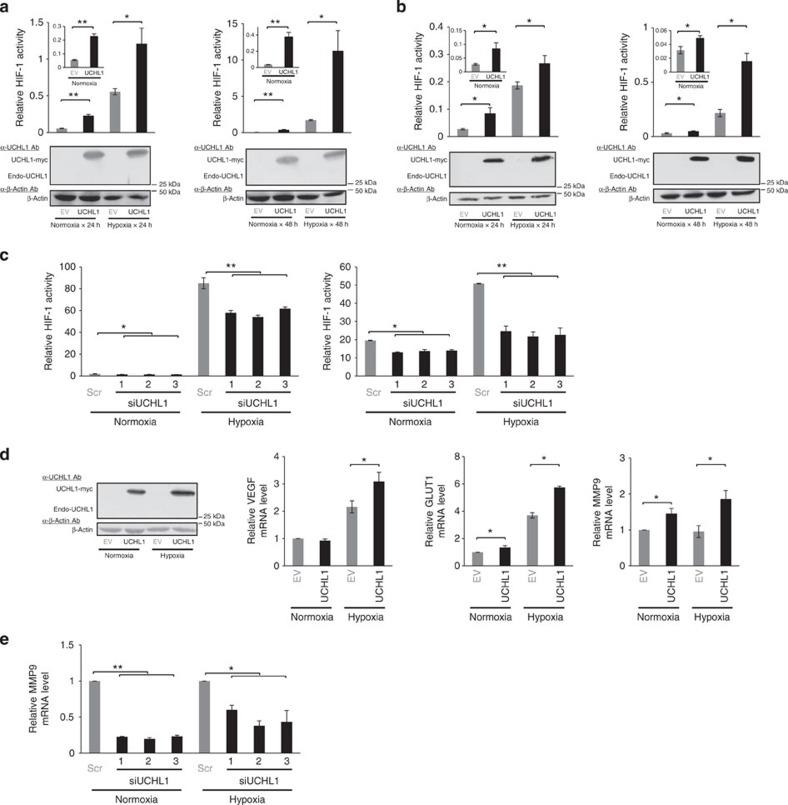
UCHL1 upregulates HIF-1 activity. (**a**,**b**) HeLa/5HRE-Luc (**a**) and MCF7/5HRE-Luc (**b**) cells were transfected with pcDNA4/UCHL1 (UCHL1) or pcDNA4/myc-His A (empty vector: EV), cultured under normoxic (20%) or hypoxic (1%) conditions for the indicated period, and subjected to the luciferase assay and western blotting using the indicated antibodies. (**c**) 293T (left) and MDA-MB-436 (right) cells transiently transfected with p5HREp-Luc were treated with scramble-siRNA (Scr) or UCHL1-siRNA (siUCHL1), cultured under normoxic (20%) or hypoxic (1%) conditions for 24 h, and subjected to the luciferase assay. (**d**,**e**) HeLa (**d**) and 293T (**e**) cells were transiently transfected with either pcDNA4/UCHL1 (UCHL1) or pcDNA4/myc-His A (EV) (**d**) or treated with either scramble-siRNA (Scr) or UCHL1-siRNA (siUCHL1) (**e**), cultured under normoxic (20%) or hypoxic (1%) conditions for 24 h, and then subjected to western blotting using the indicated antibodies and qRT–PCR to quantify the mRNA levels of the indicated genes. Mean±s.d. *n*=3. *P<0.05, **P<0.01, Student’s *t*-test (**a**,**b**,**d**) and Dunnett’s test (**c**,**e**).

We performed a luciferase assay using the *5HREp-luc* reporter gene, which expressed *firefly* luciferase under the control of 5HREp^12^,^14^, to examine whether UCHL1 induced HIF-1 activity. The forced expression of UCHL1 in cervical and breast cancer cell lines that originally showed no detectable expression of endogenous UCHL1 resulted in the upregulation of *5HREp-luc* reporter activity under both normoxic and hypoxic conditions (Fig. 1a,b and Supplementary Figs 2 and 3). On the other hand, the knockdown of robust endogenous UCHL1 expression (Supplementary Figs 2,4) resulted in the significant suppression of *5HREp-luc* reporter activity independently of the oxygen conditions (Fig. 1c and Supplementary Fig. 5). The positive impact of UCHL1 on *5HREp-luc* reporter activity was almost completely abrogated by the short interfering RNA (siRNA) treatment against the HIF-1α gene (Supplementary Fig. 6). Quantitative reverse transcription PCR (qRT–PCR) analyses for *VEGF*, *GLUT1* and *MMP9* demonstrated that the forced expression and knockdown of UCHL1 resulted in the significant induction of and reduction in the expression of HIF-1-downstream genes, respectively (Fig. 1d,e and Supplementary Figs 4 and 7). These results clearly indicated that UCHL1 upregulated HIF-1 activity in various cancer cell lines.

### UCHL1 increases the stability of HIF-1α

**Figure 2:**
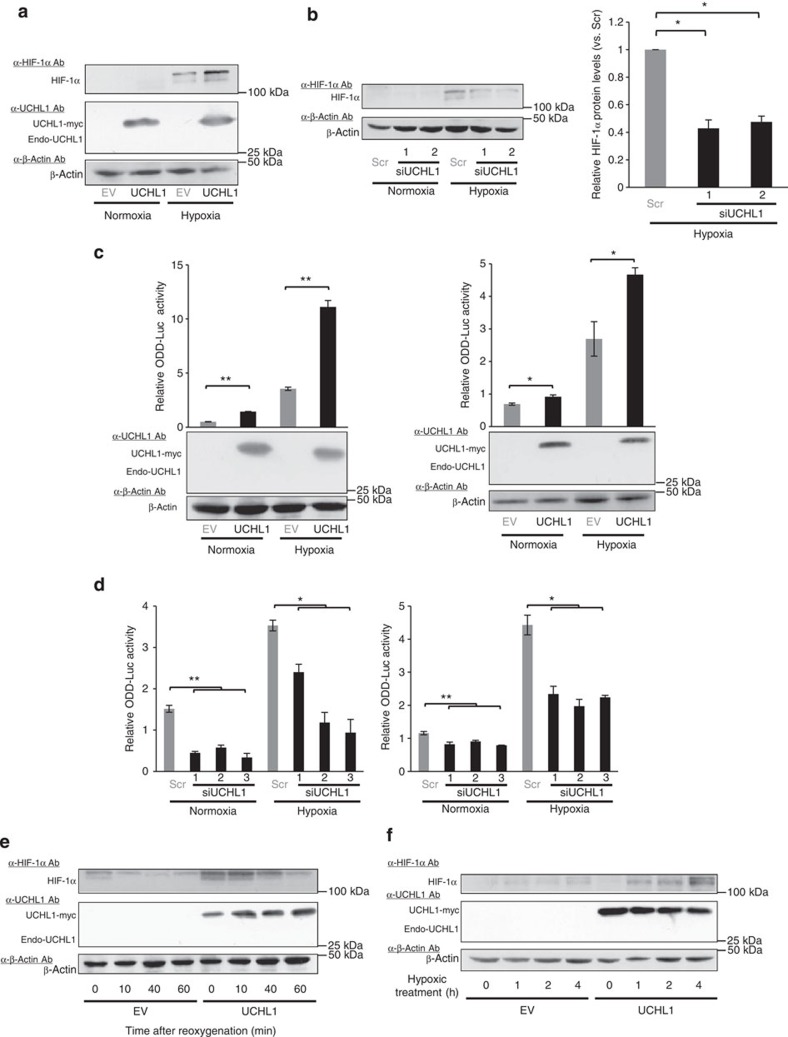
UCHL1 increases the stability of HIF-1α. (**a**,**b**) HeLa (**a**) and 293T (**b**) cells were transiently transfected with either pcDNA4/UCHL1 (UCHL1) or pcDNA4/myc-His A (EV) (**a**) or treated with either scramble-siRNA (Scr) or UCHL1-siRNA (siUCHL1) (**b**), cultured under normoxic (20%) or hypoxic (0.1%) conditions for 24 h, and then subjected to western blotting using the indicated antibodies. The intensity of the bands in the left panel of **b** was quantified and relative HIF-1α protein levels after the siUCHL1-1 or -2 treatment to those after the Scr-siRNA treatment were shown in the right panel of **b**. (**c**,**d**) HeLa/ODD-Luc (**c**: left), MCF7/ODD-Luc (**c**: right), 293T (**d**: left) and MDA-MB-436 (**d**: right) were transiently transfected with either pcDNA4/UCHL1 (UCHL1) or pcDNA4/myc-His A (EV) (**c**), or treated with either scramble-siRNA (Scr) or UCHL1-siRNA (siUCHL1) (**d**), cultured under normoxic (20%) or hypoxic (1%) conditions for 24 h, and then subjected to western blotting using the indicated antibodies and the luciferase assay. (**e**) HeLa cells were transiently transfected with pcDNA4/UCHL1 (UCHL1) or pcDNA4/myc-His A (EV), cultured under hypoxic conditions (0.1%) for 24 h, treated with cycloheximide (10 μg ml^−1^) under normoxic conditions (20%) for the indicated period, and harvested for western blotting with the indicated antibodies. (**f**) Western blotting of the HeLa cell lysate after the hypoxic treatment (1%) for the indicated periods with or without the transient transfection of the UCHL1 expression vector. Mean±s.d. *n*=3. *P<0.05, **P<0.01, Student’s *t*-test (**b**,**c**) and Dunnett’s test (**d**).

We then examined the molecular mechanisms underlying the upregulation of HIF-1 by UCHL1. UCHL1 was previously reported to function as a deubiquitinating enzyme^15^; therefore, we assumed that it would recognize HIF-1α as a substrate and increase its stability in cancer cells. As hypothesized, immunoblotting clearly demonstrated that the expression of the HIF-1α protein was significantly increased after the overexpression of UCHL1 under hypoxic conditions (Fig. 2a). However, it was difficult to observe an increase in this protein under normoxic conditions as basal HIF-1α expression levels were below detectable levels in this experimental condition (Fig. 2a). The induction of HIF-1α protein expression under low-oxygen conditions was significantly decreased when cells were treated with siRNA against the endogenous UCHL1 gene (Fig. 2b and Supplementary Fig. 4). To further determine whether UCHL1 influenced the stability of the HIF-1α protein, we used the *SV40p-ODD-luc* reporter gene, which emits luciferase bioluminescence under the same regulation as HIF-1α stability because of the fusion of luciferase to the ODD domain (HIF-1α 548–604 amino acid (a.a.))^16^. The reporter assay demonstrated that the overexpression and knockdown of UCHL1 significantly increased and decreased ODD-Luc fusion protein levels, respectively (Fig. 2c,d and Supplementary Fig. 4). Moreover, the forced expression of UCHL1 prolonged the half-life of the fusion protein following reoxygenation (Supplementary Fig. 8). Western blotting demonstrated that UCHL1 functioned not only in stabilizing the HIF-1α protein following the reoxygenation treatment (Fig. 2e), but also in the accelerated accumulation of HIF-1α after the availability of oxygen decreased (Fig. 2f). These results collectively indicated that UCHL1 positively influenced the stability of the HIF-1α protein.

### UCHL1 functions as a deubiquitinating enzyme for HIF-1α

**Figure 3:**
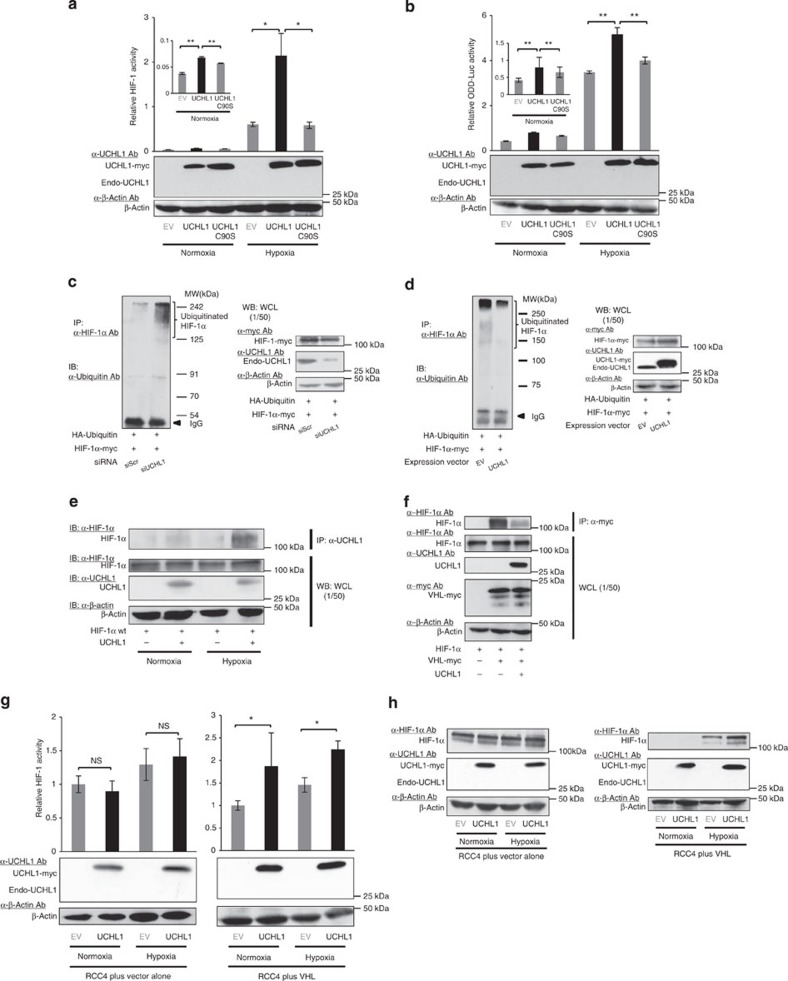
UCHL1 functions as a deubiquitinating enzyme for HIF-1α. (**a**,**b**) HeLa/5HRE-Luc (**a**) and HeLa/ODD-Luc (**b**) cells were transiently transfected with pcDNA4/UCHL1 (UCHL1), pcDNA4/UCHL1 C90S (UCHL1 C90S) or pcDNA4/myc-His A (EV), cultured under normoxic (20%) or hypoxic (1%) conditions for 24 h, and subjected to western blotting using the indicated antibodies and luciferase assay. (**c**,**d**) 293T cells transiently transfected with both pcDNA4A/HIF-1α-myc and pMT132 were treated with either scramble-siRNA (Scr) or UCHL1-siRNA (siUCHL1) (**c**) or additionally transfected with pcDNA4/myc-His A (EV) or pcDNA4/UCHL1 (UCHL1) (**d**), and cultured with MG132 (30 μg ml^−1^) for 6 h. Ubiquitinated HIF-1α was immunoprecipitated using an anti-HIF-1α antibody and detected with an anti-ubiquitin antibody (left). One-fiftieth of the whole cell lysate (WCL) was subjected to western blotting with the indicated antibodies (right). (**e**) Twenty-four hours after the transient transfection with the expression vector for HIF-1α (pcDNA4A/HIF-1α) and either the expression vector for UCHL1 (pcDNA4/UCHL1) or its empty vector, cells were precultured under normoxic (20%) or hypoxic (1%) conditions for 24 h, treated with MG132 (30 μM) under the same oxygen conditions as the preculture, and subjected to immunoprecipitation with the anti-UCHL1 antibody and subsequent western blotting with the indicated antibodies. Paraformaldehyde solution (1%) was used as a cross-linking reagent. One-fiftieth of the WCL was subjected to western blotting with the indicated antibodies. (**f**) Twenty-four hours after the transient transfection with the expression vectors for HIF-1α (pcDNA4/HIF-1α), VHL-myc (pEF6/VHL-myc), UCHL1 (pcDNA4/UCHL1) or their empty vector, cells were treated with MG132 (30 μg ml^−1^) for an additional 6 h. VHL-myc was immunoprecipitated using an anti-myc antibody and co-precipitated HIF-1α was detected. One-fiftieth of the WCL was subjected to western blotting with the indicated antibodies (lower). (**g**,**h**) RCC4 plus vector alone and RCC4 plus VHL cells were transiently transfected with p5HREp-luc and either pcDNA4/UCHL1 (UCHL1) or pcDNA4/myc-His A (EV), cultured under normoxic (20%) or hypoxic (0.1%) conditions for 24 h, and subjected to western blotting using the indicated antibodies and luciferase assay. Mean±s.d. *n*=3. *P<0.05; NS, not significant, Student’s *t*-test (**g**) and Dunnett’s test (**a**,**b**). IP, immunoprecipitation; IB, immunoblot; WB, western blotting.

We then investigated whether the deubiquitinating activity of UCHL1 played an important role in increasing the stability of HIF-1α and activity of HIF-1. We constructed a plasmid expressing the catalytically inactive mutant form of UCHL1, in which a cysteine residue, C_90_, was substituted for serine. The C90S mutation abrogated the UCHL1-mediated increase in both *5HREp-luc* and *SV40p-ODD-luc* reporter activities under normoxic and hypoxic conditions (Fig. 3a,b). Moreover, when the ubiquitination status of the HIF-1α protein was directly evaluated by performing immunoprecipitation assays, the knockdown of endogenous UCHL1 significantly increased HIF-1α ubiquitination (Fig. 3c and Supplementary Fig. 9). On the other hand, the overexpression of UCHL1 resulted in a decrease in HIF-1α ubiquitination (Fig. 3d). The deubiquitinating effect on HIF-1α appeared to have been achieved by a direct interaction between UCHL1 and HIF-1α (Fig. 3e). Moreover, this interaction was confirmed to prevent VHL from directly interacting with HIF-1α (Fig. 3f). Furthermore, luciferase assays using the *5HREp-luc* reporter gene demonstrated that the overexpression of UCHL1 had no impact on HIF-1 activity in VHL-defective RCC4 cells (RCC4 plus vector alone; Fig. 3g, left), but had a positive influence in VHL-reconstituted RCC4 cells (RCC4 plus VHL; Fig. 3g, right). Western blotting consistently showed that the UCHL1-dependent increase in HIF-1α protein levels was only observed in the presence of VHL (Fig. 3h). Our results indicated that the aberrant expression of UCHL1 in cancer cells abrogated the VHL-mediated ubiquitination of HIF-1α, which led to the stabilization of HIF-1α and subsequent activation of HIF-1.

### UCHL1 promotes distant tumour metastases

**Figure 4:**
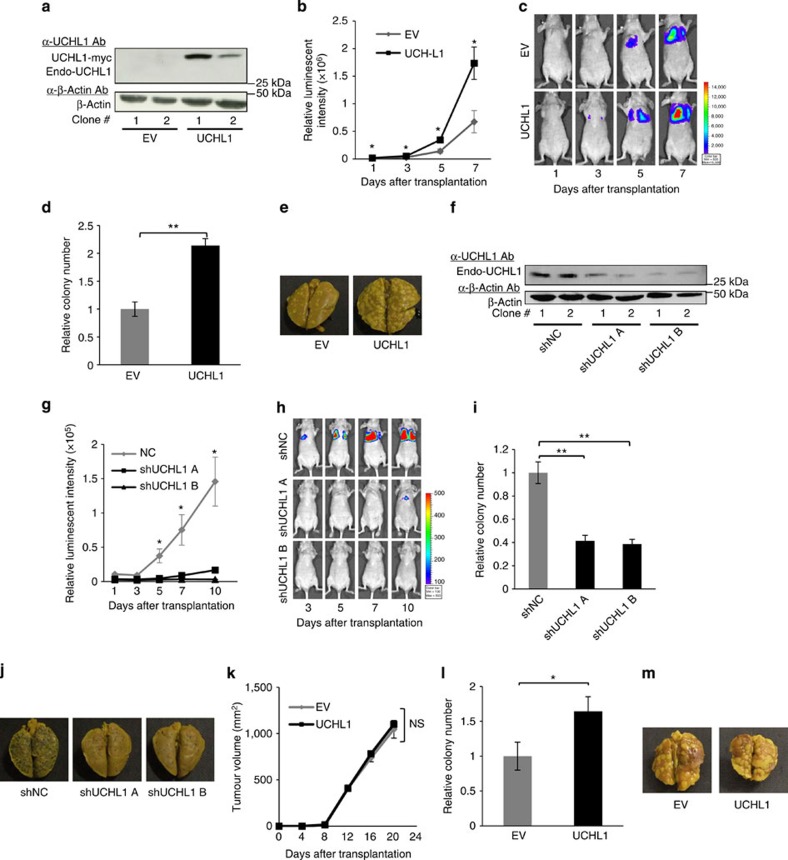
UCHL1 promotes distant tumour metastases. (**a**) Two clones of the EMT6/EF-Luc/EV and EMT6/EF-Luc/UCHL1 cells were subjected to western blotting with the indicated antibodies. (**b**–**e**) The growth of pulmonary metastases was monitored by *in vivo* imaging as luciferase bioluminescence on the indicated days after the intravenous transplantation of EMT6/EF-Luc/EV (EV) and EMT6/EF-Luc/UCHL1 (UCHL1) cells (**b**). (**c**) Representative images. (**d**) The relative number of metastatic lung colonies in the UCHL1 group to that in the EV group was analysed 10 days after transplantation. (**e**) Representative images. *n*=12. (**f**–**j**) By using two clones of B16F10/EF-Luc/shNC and B16F10/EF-Luc/shUCHL1 (shRNA version A and B) cells (**f**), the same experiments as those described in **a** to **e** were performed. (**k**–**m**) EMT6/EF-Luc/EV (EV) and EMT6/EF-Luc/UCHL1 (UCHL1) cells were orthotopically transplanted to the fat pads of female scid mice. The growth of primary tumours (**k**) and relative number of metastatic lung colonies in the UCHL1 group to that in the EV group was analysed 27 days after transplantation (**l**). *n*=6. (**m**) Representative images. Mean±s.d. *P<0.05, **P<0.01, Student’s *t*-test (**b**,**d**,**g**,**i**,**k**,**l**). NS, not significant.

We subsequently investigated the influence of the UCHL1-mediated activation of HIF-1 on tumour malignancy. We focused on metastasis as we already determined that UCHL1 induced the expression of metastasis-related genes, such as *MMP2* and *MMP9* (Fig. 1d,e and Supplementary Fig. 7). We used a murine model of pulmonary metastasis, in which a cancer cell suspension was intravenously injected into the tail vein of immunodeficient mice, and the number of resultant metastatic colonies was externally quantified in the lungs. To monitor the growth of pulmonary metastases in real-time, we utilized the stable transfectants of cancerous EMT6 and B16F10 cells with the pEF-luc plasmid^12^, which constitutively expressed *firefly* luciferase (EMT6/EF-Luc and B16F10/EF-Luc, respectively). EMT6/EF-Luc cells, which originally expressed negligible amounts of endogenous UCHL1 (Supplementary Fig. 2), were stably transfected with the UCHL1 expression vector or its empty vector (Fig. 4a). The resultant clones, EMT6/EF-Luc/UCHL1 and EMT6/EF-Luc/EV cells, showed that the forced expression of UCHL1 induced the expression of HIF-1α and HIF-1 activity (Supplementary Fig. 10a,b) and enhanced cell migration (Supplementary Fig. 11), but did not promote cell proliferation *in vitro*, EF-Luc reporter activity or the growth of tumour xenografts following subcutaneous transplantation (Supplementary Fig. 10c–e). Optical imaging after the intravenous transplantation of these cells in the mouse model of pulmonary metastasis showed that the overexpression of UCHL1 significantly accelerated the increase in bioluminescent intensity in the lungs (Fig. 4b,c). The number of metastatic colonies was markedly increased by the forced expression of UCHL1 (Fig. 4d,e). These results suggested that UCHL1, possibly through the activation of HIF-1, promoted the formation of metastatic colonies without accelerating the proliferation of cancer cells. This conclusion was supported by a loss-of-function study on the murine model of pulmonary metastasis using the murine melanoma cell line, B16F10, which originally showed the higher endogenous expression of UCHL1 (Supplementary Fig. 2). We used B16F10/EF-Luc cells stably transfected with the plasmid expressing either short-hairpin RNA (shRNA) for mouse UCHL1 or scramble shRNA (as a negative control) to establish B16/EF-Luc/shUCHL1 and B16/EF-Luc/shNC, respectively. Although the silencing of endogenous UCHL1 (Fig. 4f and Supplementary Fig. 12a,b) did not inhibit EF-Luc reporter activity or cell proliferation *in vitro* (Supplementary Fig. 12c,d), it did inhibit the induction of HIF-1α expression under hypoxic conditions (Supplementary Fig. 12b) and eventually suppressed the growth and formation of pulmonary metastasis (Fig. 4g–j).

We next used a complementary murine model of pulmonary metastasis, in which the stable transfectants of the murine breast cancer cells, EMT6, with the UCHL1 expression vector or its empty vector were transplanted orthotopically into the mammary fat pad (Fig. 4k–m). Although the overexpression of UCHL1 did not promote the growth of primary tumour xenografts in the mammary fat pad (Fig. 4k), the number of metastatic colonies was markedly increased by the forced expression of UCHL1 (Fig. 4l,m). This result further strengthened the conclusion that UCHL1 promoted distant tumour metastases.

### UCHL1 promotes metastases in a HIF-1-dependent manner

**Figure 5:**
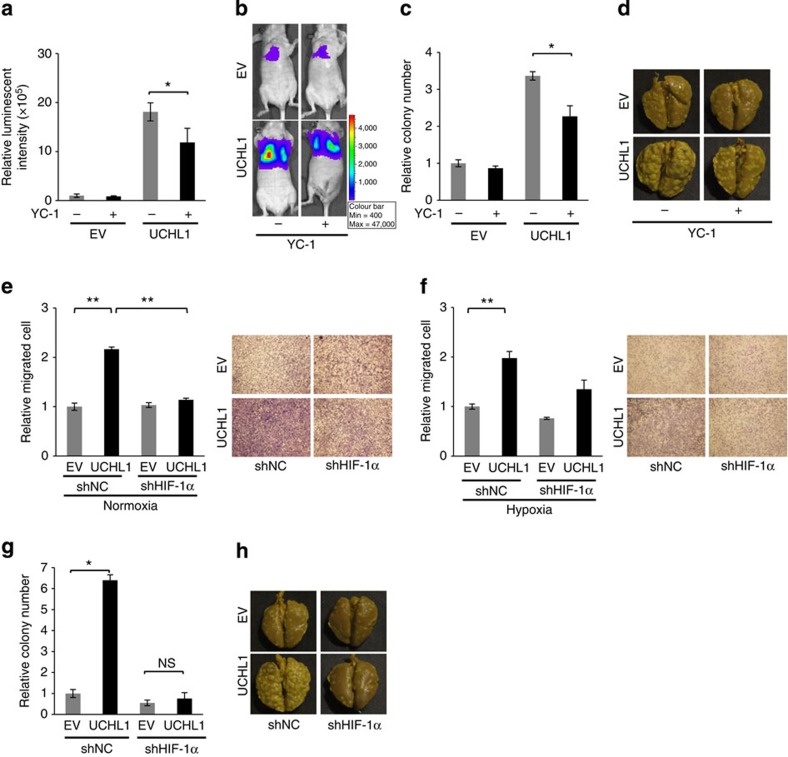
UCHL1-mediated promotion of distant tumour metastases are HIF-1 dependent. (**a–d**) Mice were treated with (+) or without (−) the HIF-1 inhibitor, YC-1 (30 mg kg^−1^ daily) between 3 and 5 days after the intravenous transplantation of EMT6/EF-Luc/EV (EV) and EMT6/EF-Luc/UCHL1 (UCHL1) cells. (**a**) The total volume of pulmonary metastases was monitored by *in vivo* imaging as luciferase bioluminescence 7 days after transplantation. *n*=4. (**b**) Representative images. (**c**) Relative number of metastatic lung colonies to the EV and YC-1 (−) group. *n*=4. (**d**) Representative images. (**e**,**f**) A Transwell migration assay using EMT6/EF-Luc/shNC/EV (upper left in the right panels in **e** and **f**), EMT6/EF-Luc/shHIF-1α/EV (upper right), EMT6/EF-Luc/shNC/UCHL1 (lower left) and EMT6/EF-Luc/shHIF-1α/UCHL1 (lower right) cells under normoxic (20%, **e**) and hypoxia (1%, **f**) conditions. Mean±s.d. *n*=4, *P<0.05. Representative images are shown on the right. (**g**,**h**) By using two clones of EMT6/EF-Luc/shNC/EV, EMT6/EF-Luc/shHIF-1α/EV, EMT6/EF-Luc/shNC/UCHL1 and EMT6/EF-Luc/shHIF-1α/UCHL1 cells, the same experiments as those described in Fig. 4d,e were performed. (**g**) Relative number of metastatic lung colonies to the EV and shNC group. *n*=12. Mean±s.d. *P<0.05, **P<0.01; NS, not significant, Student’s *t*-test (**a**,**c**,**e**,**f**,**g**).

We used an inhibitor of HIF-1, YC-1, and a plasmid expressing shRNA for HIF-1α, shHIF-1α, to directly examine the involvement of HIF-1α in the formation of pulmonary metastasis enhanced by UCHL1. Mice were treated daily with YC-1 from 3 to 5 days after the intravenous transplantation of EMT6/EF-Luc/EV and EMT6/EF-Luc/UCHL1 cells. The YC-1 treatment significantly decreased the UCHL1-dependent increase in bioluminescent intensity and reduced the number of metastatic colonies in the lungs (Fig. 5a–d). We then performed experiments using EMT6/EF-Luc/shHIF-1α cells that were stably transfected with both the UCHL1-expressing vector and HIF-1α-silencing vector (Supplementary Fig. 13). HIF-1α silencing was confirmed to suppress the UCHL1-mediated promotion of cell migration in a Transwell migration assay (Fig. 5e,f). The experimental metastasis assay confirmed that stable HIF-1α knockdown almost completely abrogated the UCHL1-mediated increase in the number of metastatic foci (Fig. 5g,h). These results collectively demonstrated that the overexpression of UCHL1 promoted distant tumour metastases in a HIF-1-dependent manner.

### Prognostic value of UCHL1 expression level in human cancer

**Figure 6:**
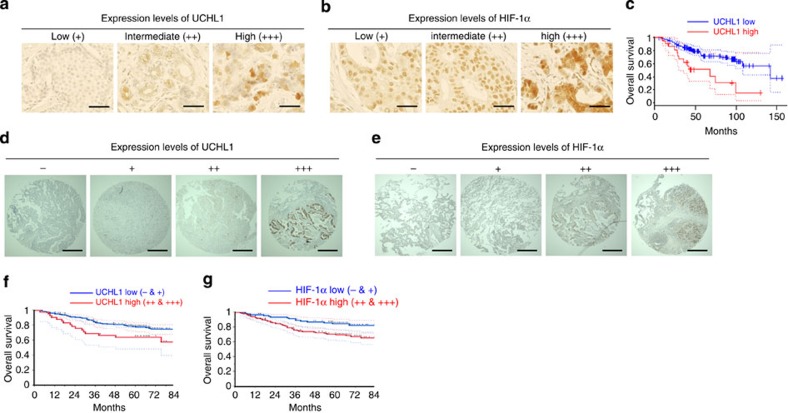
UCHL1 is a good prognostic marker. (**a**–**c**) Human breast tumour sections (30 samples) were stained with anti-UCHL1 (**a**) and anti-HIF-1α (**b**) antibodies (scale bar, 50 μm). (**c**) A PrognoScan database-based Kaplan–Meier analysis of the overall survival of 158 breast cancer patients stratified by high (red) and low (blue) UCHL1 levels (high: *n*=23, low: *n*=135; log-rank tests *P*<0.05). (**d**–**g**) Microarrays spotted with human lung tumour tissues (254 samples) were stained with anti-UCHL1 (**d**) and anti-HIF-1α (**e**) antibodies (scale bar, 500 μm). A Kaplan–Meier analysis of the overall survival of the 254 lung cancer patients stratified by high (red; +++ and ++ in **d**,**e**) and low (blue;+and −in **d**,**e**) UCHL1 (**f**) and HIF-1α (**g**) levels (log-rank tests, *P*<0.05).

To validate our results in human tumours, we first performed an immunohistochemical analysis on human breast cancers using anti-UCHL1 and anti-HIF-1α antibodies (Fig. 6a,b and Supplementary Figs 14,15). Tumour samples were categorized into three groups (high, intermediate and low) according to the expression levels of UCHL1 and HIF-1α, respectively. We confirmed that the expression levels of UCHL1 positively correlated with those of HIF-1α (Table 1; *R*^*2*^=0.534). We then analysed the relationship between UCHL1 expression levels and poor prognosis using the PrognoScan database. We confirmed that the expression levels of UCHL1 positively correlated with lower overall survival and distant metastasis-free survival rates in human breast cancer, lung cancer and melanoma (Fig. 6c and Supplementary Fig. 16). We next performed the same type of analyses on tissue microarrays mounted with human lung cancers^17^,^18^. Tumour samples were categorized into four groups (+++, ++, +, −) according to the expression levels of UCHL1 and HIF-1α, respectively. The expression levels of UCHL1 correlated well with those of HIF-1α in lung tumours (Fig. 6d,e and Table 2). Moreover, high expression levels of both UCHL1 and HIF-1α (+++ and ++) were strongly associated with the poor overall survival of lung cancer patients (Fig. 6f,g). These results suggested that the expression of UCHL1 would lead to a poor prognosis by activating HIF-1; therefore, it could be a useful prognostic marker.

**Figure 7:**
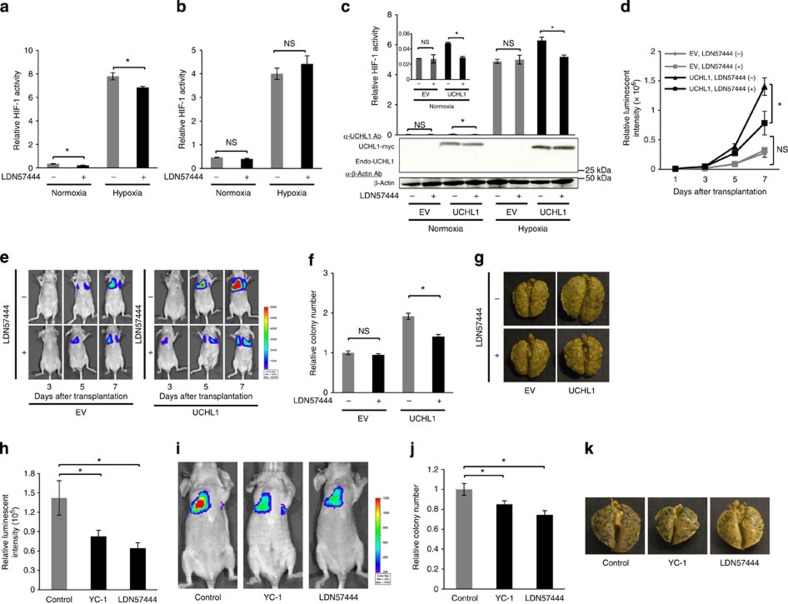
UCHL1 is a good therapeutic target. (**a**–**c**) 293T cells transiently transfected with the *5HREp-luc* reporter gene (**a**) and HeLa/5HRE-Luc cells (**b**,**c**) with none (**b**) or either pcDNA4/UCHL1 (UCHL1) or pcDNA4/myc-His A (EV) (**c**) were treated with (+) or without (−) the UCHL1 inhibitor, LDN57444, cultured under normoxic or hypoxic conditions for 24 h, and subjected to western blotting using the indicated antibodies and luciferase assay. *n*=3. (**d**–**g**) Athymic nude mice were intravenously transplanted with EMT6/EF-Luc/EV or EMT6/EF-Luc/UCHL1 cells, and treated with or without LDN57444 (0.5 mg kg^−1^ daily) from 3 to 5 days after the transplantation. (**d**,**e**) On the indicated days after transplantation, mice were subjected to an optical *in vivo* imaging experiment. *n*=6. (**f**,**g**) The relative number of metastatic colonies to the EV and LDN57444 group was analysed in the lungs 10 days after transplantation. *n*=6. (**h**–**k**) Athymic nude mice were transplanted with B16F10/EF-Luc cells through the tail vein, and treated with or without YC-1 (30 mg kg^−1^ daily) or LDN57444 (0.5 mg kg^−1^ daily) from 3 to 5 days after transplantation. (**h**,**i**) Mice were subjected to an optical *in vivo* imaging experiment 7 days after transplantation. *n*=9. (**j**,**k**) The relative number of metastatic colonies to the control group was analysed in the lungs 10 days after transplantation. *n*=9. Mean±s.d. *P<0.05; NS, not significant, Student’s *t*-test (**a**–**d**,**f**,**h**,**j**).

We used the small-molecule UCHL1 inhibitor, LDN57444, to establish whether UCHL1 could be exploited as a therapeutic target to reduce the incidence of distant tumour metastases. Using the *5HREp-luc* reporter gene *in vitro*, we confirmed that LDN57444 significantly inhibited HIF-1 activity in the presence of endogenous UCHL1 expression in 293T cells (Fig. 7a); however, it had no effect on HIF-1 activity in HeLa cells because of the absence of endogenous UCHL1 (Fig. 7b). The reconstitution of UCHL1 in HeLa cells restored the HIF-1-blocking activity of the drug (Fig. 7c). The *in vivo* murine model of pulmonary metastasis also demonstrated that LDN57444 did not inhibit metastatic tumour formation when the endogenous expression of UCHL1 was low (Fig. 7d,e, EV), but did when UCHL1 was reconstituted (Fig. 7d,e, UCHL1). The number of metastatic colonies 10 days after the intravenous transplantation of EMT6/EF-Luc/EV and EMT6/EF-Luc/UCHL1 cells also revealed that LDN57444 had an antimetastatic effect on UCHL1-expressing cancer cells (Fig. 7f,g). LDN57444 was not less effective than YC-1 in inhibiting pulmonary metastasis (Fig. 7h–k). Although UCHL1 was previously reported to be expressed in neurons, testis and ovary, we did not observe any obvious side effects after the administration of LDN57444, such as reductions in body weights or behavioural disorders, at least in our experimental setting (Supplementary Figs 17 and 18a). Furthermore, the administration of LDN57444 to both male and female mice during their mating was not confirmed to cause a decrease in the body weights of neonatal mice (Supplementary Fig. 18b). Taken together, these results indicated that UCHL1 is a good therapeutic target for the suppression of distant tumour metastases.

## Discussion

In the present study, we provided a novel insight into the regulatory mechanism underlying tumour metastasis via activation of the UCHL1-HIF-1 pathway. It has been widely accepted that HIF-1 functions in multiple steps of distant tumour metastasis^3^,^7^, for example, in the acquisition of an invasive phenotype of cancer cells through the so-called epithelial-to-mesenchymal transition, local invasion to blood vessels by degrading the extracellular matrix, formation of a premetastatic niche in distant organs, extravasation by degrading the extracellular matrix and adaptation to the microenvironments of distant tissues^3^,^19^. UCHL1 has also been shown to be a key regulator of the invasion and metastasis of cancer cells^20^; however, its downstream factors have yet to be characterized. Under these conditions, one of the most significant advancements of this study is the mechanistic and functional link detected between UCHL1 and HIF-1 in distant tumour metastasis. This result as well as recent clinical findings, in which the expression levels of HIF-1α and UCHL1 correlated with the tumour stage, grading and overall survival of breast cancer patients^4^,^5^,^21^, offers a rational basis for targeting the UCHL1–HIF-1 axis (deubiquitination of HIF-1α) to control cancer metastases.

In the present study, we provided direct evidence to show that UCHL1 was responsible for the deubiquitination of HIF-1α and upregulated HIF-1 activity. To date, UCHL1 has been reported to exhibit ubiquitin hydrolase activity^22^; however, the influence of UCHL1 on HIF-1α has not yet been elucidated. The factors reported as the downstream substrates of UCHL1 were the pro-apoptotic gene, NOXA, and the mediator of Wnt signalling, β-catenin^23^,^24^; on the other hand, the gene that was shown to deubiquitinate HIF-1α was pVHL-interacting deubiquitinating enzyme 2 (VDU2)^25^. Although the detailed mechanisms responsible for the actions of and relationship between UCHL1 and VDU2 remain unclear, this study emphasized the importance of the deubiquitination of HIF-1α, in addition to the increase in the stability and transactivation activity of HIF-1α, in the positive regulation of HIF-1 activity. To fully elucidate the physiological and pathophysiological importance of deubiquitination, it is critical to more thoroughly analyse the meaning of our immunoprecipitation data in which UCHL1 bound to HIF-1α and prevented VHL from directly interacting with HIF-1α (Fig. 3e,f) and the interaction between UCHL1 and HIF-1α was facilitated under hypoxic conditions (Fig. 3e).

The importance of UCHL1 in the stabilization of HIF-1α and resultant activation of HIF-1 was also validated in clinical human tumours; the expression levels of UCHL1 correlated well with those of HIF-1α in both breast and lung cancers. However, the influence of UCHL1 on HIF-1α expression observed in the present study appeared to be greater in clinical tumour tissues than in cancer cells cultured under simple low-oxygen conditions. Although the reason for this difference is currently unclear, one possibility is that tumour-specific microenvironments other than simple hypoxia, such as a decrease in the availability of glucose as well as oxygen, may be important for the activity of the UCHL1–HIF-1 axis based on the results of our *in vitro* experiment; the impact of UCHL1 on HIF-1α stability was facilitated when glucose concentrations decreased under hypoxic conditions (Supplementary Fig. 19).

We confirmed that UCHL1 expression levels in malignant tumours correlated with the poor prognosis of patients with breast and lung cancers. This result was consistent with the findings of previous studies in which UCHL1 was shown to be involved in tumour metastases^20^,^26^ and closely associated with the advanced stages of lung cancer as well as with the tumorigenesis, progression and invasiveness of cancers^27^,^28^,^29^. These findings have justified exploiting UCHL1 as a prognostic marker and treatment target for cancers. However, in order to realize diagnostic and therapeutic strategies, it is critical to approach the following issues: (i) the influence of UCHL1 not only on HIF-1α, but also on HIF-2α, (ii) the factors required for the recognition of downstream substrates by UCHL1 and (iii) the expression levels of UCHL1 in normal tissues. Furthermore, as epigenetic alterations have been suggested to be important for regulating the expression and functions of UCHL1 (refs 30, 31, 32, 33), it is also important to analyse their meaning and underlying mechanism in more detail. Potential side effects following the systemic administration of UCHL1 inhibitors also need to be examined and drug delivery systems have to be developed for these inhibitors as UCHL1 expression has been confirmed to some extent in neurons, the testis and ovary. Solutions to these issues should lead to the development of novel antimetastatic strategies in the future.

## Methods

### Cell culture and reagents

NIH/3T3, HeLa, MCF7, MDA-MB-231, MDA-MB-436, DU 145, 293T, EMT6 and B16F10 were purchased from the American Type Culture Collection. RCC4 plus vector alone and RCC4 plus VHL, which are human renal cell carcinoma cell lines (RCC4) stably transfected with pcDNA3 (empty vector) and pcDNA3-VHL (VHL-expressing vector), were purchased from DS Pharma Biomedical. Cells were maintained in 10% FBS-Dulbecco’s modified Eagle’s medium. Cells were incubated in a well-humidified incubator with 5% CO_2_ and 95% air for the normoxic incubation or in the Bactron Anaerobic Chamber, BACTLITE-2 (Sheldon Manufacturing) and in a RUSKIN INVIVO_2_ 500 (Ruskinn) for the hypoxic incubation at <0.1% O_2_ and 1% O_2_, respectively. Stock solutions of cycloheximide (Nacalai Tesque) and LDN57444 (Merck) were prepared in dimethylsulphoxide (10 mg ml^−1^ and 10 mM, respectively). Double-stranded RNAs for the transient silencing of UCHL1 (silencer Select Validated siRNA, Cat# 4390824-s14616: 5′-AAGUUAGUCCUAAAGUGUATT-3′, 4390824-s14617: 5′-GCACAAUCGGACUUAUUCATT-3′, 4390824-s14618: 5′-GACCAUUGGGAAUUCCUGUTT-3′) and for the negative control (Cat# 12935-300: sequence information is not disclosed) were purchased from Life Technologies.

### Plasmid constructs

To construct pcDNA4/UCHL1, the cDNA encoding human *uchl1* gene was amplified from the cDNA of HeLa cells and inserted between the *Eco*RV and *Xho*I sites of pcDNA4/myc-His A (Invitrogen). To construct pcDNA4/UCHL1 C90S, the C90S mutation was introduced into pcDNA4/UCHL1 using PCR-based site-directed mutagenesis with the following primers; 5′-AATTCCTCTGGCACAATCGGACTTATTC-3′ and 5′-CGATTGTGCCAGAGGAATTCCCAATGG-3′. To construct p5HRE-BSD, the *luciferase* coding sequence between the *Nco*I and *Xba*I sites of p5HRE-Luc^12^ was substituted for the *bsd*-coding fragment. To construct pEF/SV40p-ODD-Luc, the DNA fragment encoding the *SV40p-ODD-luc* reporter gene was prepared by digesting pGL3/ODD-Luc with *Kpn*I and *Xba*I, and inserted between the corresponding sites of pEF/myc/cyto (Invitrogen). The plasmids, pEF-Luc and p5HRE-Luc, were constructed as described previously^12^. The haemagglutinin (HA)-tagged ubiquitin expression plasmid, pMT123, was described previously^34^. To construct the plasmid pcDNA4/HIF-1α-myc and pcDNA4/HIF-1α, the coding sequence of the HIF-1α gene was amplified by PCR from the human cDNA library and inserted between the *Bam*HI and *Eco*RV sites of pcDNA4/myc-His B and pcDNA4/myc-His A (Invitrogen), respectively. To construct pEF6/VHL-myc, the cDNA encoding the human *vhl* gene was inserted between the *Eco*RI and *Xba*I sites of pEF6/myc-His A (Invitrogen). Plasmids expressing shRNA for mouse UCHL1 (Cat# 336314 KM27639P, clone A insert sequence: 5′-CGAAGATAGAGCCAAGTGTTT-3′ and clone B insert sequence: 5′-TGAAGCAGACCATCGGAAACT-3′), for human HIF-1α (Cat# 336314 KM03799P, clone A insert sequence: 5′-ATAGCGATATGGTCAATGTAT-3′ and clone B insert sequence: 5′-CGAGTGAAAGGATTCATATCT-3′) and for the negative control (Cat# 336314 KM27639P, NC insert sequence: 5′-GGAATCTCATTCGATGCATAC-3′) were purchased from QIAGEN.

### Stable transfectants

NIH3T3, HeLa, MCF7, MDA-MB-231, EMT6 or B16F10 cells were stably transfected using the calcium phosphate method^35^ with p5HRE-BSD (for NIH3T3/5HRE-BSD), pEF/SV40p-ODD-Luc (for HeLa/ODD-Luc and MCF7/ODD-Luc), p5HRE-Luc (for HeLa/5HRE-Luc, MCF7/5HRE-Luc, MDA-MB-231/5HRE-Luc, EMT6/5HRE-Luc and B16F10/5HRE-Luc), pEF-Luc (for EMT6/EF-Luc and B16F10/EF-Luc), the combination of pEF-Luc and either pcDNA4/myc-His A (Invitrogen; for EMT6/EF-Luc/EV (clone #1, 2)) or pcDNA4/UCHL1 (for EMT6/EF-Luc/UCHL1 (clone #1, 2)), the combination of pEF-Luc and the plasmid expressing either shRNA for mouse UCHL1 (QIAGEN; 336314 KM27639P; for B16F10/EF-Luc/shUCHL1 (clone #A1, A2, B1 and B2)) or the negative control (QIAGEN; 336314 KM27639P; for B16F10/EF-Luc/shNC (clone #1, 2)), or the combination of pEF-Luc and either the plasmid expressing either shRNA for mouse HIF-1α (QIAGEN; 336314 KM03799P; for EMT6/EF-Luc/shHIF-1α (clone #1, 2)) or the negative control (QIAGEN; 336314 KM03799P; for EMT6/EF-Luc/shNC (clone #1, 2)). EMT6/EF-Luc/shHIF-1α cells were stably transfected with either pcDNA4/myc-His A (for EMT6/EF-Luc/shHIF-1α/EV (clone #1, 2)) or pcDNA4/UCHL1 (for EMT6/EF-Luc/shHIF-1α/UCHL1 (clone #1, 2)). EMT6/EF-Luc/shNC cells were stably transfected with either pcDNA4/myc-His A (for EMT6/EF-Luc/shNC/EV (clone #1, 2)) or pcDNA4/UCHL1 (for EMT6/EF-Luc/shNC/UCHL1 (clone #1, 2)). Cells were cultured for 12–14 days in culture medium containing the corresponding antibiotic to select antibiotic-resistant stable transfectants. The resultant colonies were isolated and established as clones. Representative clones showing the expected and reasonable activities were used in the present study.

### Screening of upstream activators of HIF-1

EcoPack2-293 cells (BD Bioscience; 2 × 10^6^ cells per φ100 mm dish), which were HEK-293-based packaging cells, were transiently transfected with 20 μg of the plasmid encoding the human placenta cDNA library (Clontech) for retrovirus production. NIH3T3/5HRE-BSD cells were infected with the resultant retroviruses, and cultured in the presence of 5 μg ml^−1^ blasticidin S under normoxic conditions for 10 days. DNA fragments encoding the potential activators of HIF-1 were rescued by PCR using 5′ and 3′ LIB primers (Clontech) from the genomic DNA of surviving colonies and then subjected to the sequencing analysis.

### Luciferase assay and western blotting

Twenty-four hours after cells (1 × 10^4^ cells per well in a 24-well plate for the luciferase assay and 2 × 10^5^ cells per well in a 6-well plate for western blotting) were transfected with the indicated plasmids, they were treated under normoxic (20% O_2_) and hypoxic (0.1 or 1% O_2_) conditions for the indicated periods, and harvested in 100 μl Passive Lysis Buffer (Promega) for the luciferase assay or 150 μl Cell Lytic Buffer (Sigma-Aldrich) for western blotting. The luciferase assay was performed using the dual luciferase assay kit according to the manufacturer’s instructions (Promega). The plasmid, pRL-SV40 (Promega), was used as an internal control to calculate relative luciferase activity. Western blotting was performed using anti-HIF-1α (500-fold dilution, BD Bioscience for human HIF-1α; 500 times dilution, Novus Biologicals for mouse HIF-1α), anti-UCHL1 (500-fold dilution, Sigma-Aldrich), anti-myc epitope tag (1,000-fold dilution, Cell Signaling) and anti-β-actin (500-fold dilution, BioVision) antibodies as the primary antibodies, anti-mouse and anti-rabbit IgG horseradish peroxidase-linked whole antibodies (5,000-fold dilution; GE Healthcare) as the secondary antibodies, and the ECL Plus Western Blotting Detection system (GE Healthcare) for detection according to the manufacturer’s instructions. Images of bands were acquired using a digital photo scanner GT-X750 (Seiko Epson Corp) and quantified using ImageJ as described previously^36^. Images have been cropped for presentation. Full size images are presented in Supplementary Fig. 20.

### qRT–PCR

Twenty-four hours after cells (2 × 10^5^ cells per well in a 6-well plate) were transfected with the indicated plasmids, total RNA was extracted using Sepasol RNA I Super according to the manufacturer’s instructions (Nacalai Tesque). Total RNA (1 μg) was subjected to reverse transcription using RNA LA PCR Kit (AMV) version 1.1 (Takara Bio). The mRNA levels of the indicated genes were quantified using the qRT–PCR technique with the Thermal Cycler Dice Real Time System (TP-800; Takara Bio) with the SYBR Premix Ex Taq kit (Takara Bio) and primers (Takara Primer Set ID HA160790 for VEGFA mRNA, HA126121 for GLUT1 mRNA, HA173965 for MMP2 mRNA, HA129244 for MMP9 mRNA, HA162299 for human UCHL1 mRNA, MA116539 for mouse UCHL1 mRNA, HA067803 for human β-actin mRNA and MA050368 for mouse β-actin mRNA) according to the manufacturer’s instructions. β-Actin mRNA levels were used as a normalizer.

### Immunoprecipitation assay

Twenty-four hours after cells (1.6 × 10^6^ cells per 100 mm dish) were transfected with the indicated plasmids and/or with the indicated siRNA, they were harvested in 1,000 μl Cell Lytic Buffer (Sigma-Aldrich), and immunoprecipitated using the Immunoprecipitation Kit Dynabeads Protein G (Life Technologies) with the indicated antibody according to the manufacturer’s instructions. Western blotting was performed using the indicated antibody.

### Transwell migration assay

Cells were seeded to insert wells of the Transwell chamber (5 × 10^4^ cells per an insert well; Corning, 8-μm pore, 24-well plate format), pre-treated with serum-starved DMEM medium for 24 h and cultured under normoxic (20% O_2_ for 24 h) or hypoxic (1% O_2_ for 12 h) conditions in DMEM medium supplemented with and without FBS in bottom wells and upper insert wells, respectively, according to the manufacturer’s instructions. Migrating cells were stained with Giemsa solution.

### ***In vivo*** **experiments**

Cancer cell suspensions were transplanted into the tail veins of athymic nude mice (BALB/c nu/nu; Japan SLC Inc., 3 × 10^4^ cells per mouse) or into the mammary fat pads of scid mice (C.B-17/Icr-scid/scid Jcl, CLEA Japan, Inc., 1 × 10^5^ cells per mouse) for a mouse model of pulmonary metastasis after intravenous or orthotopic transplantation, respectively. Optical *in vivo* imaging was performed with IVIS-SPECTRUM (Caliper)^12^,^37^. Bioluminescent images were acquired 10 min after the intraperitoneal injection of the luciferin solution (100 μl of 10 mg ml^−1^ solution). Mice were anaesthetised with 2.5% isoflurane gas in the oxygen flow (1.5 ml min^−1^) during imaging. The signal intensity was quantified and analysed using Living Image 2.50-Igor Pro 4.90 software (Caliper).

### Immunohistochemical analysis

Formalin-fixed and paraffin-embedded sections of human breast tumours were subjected to immunohistochemical staining using the DAKO LSAB+System-HRP kit (DAKO) with anti-UCHL1 (Sigma-Ardrich) and anti-HIF-1α (Novus Biologicals) antibodies as the primary antibodies, according to the manufacturer’s instructions.

### Prognoscan analysis

The relationship between UCHL1 expression levels and overall survival and distant metastasis-free survival rates was evaluated in cancer patients by the minimum *P*-value approach using the PrognoScan database^38^. Briefly, patients were divided into two groups according to UCHL1 expression levels in their tumours at all possible cutoff points. The risk differences of any two groups were then calculated by the log-rank test. The cutoff point giving the most significant *P*-value was selected and demonstrated in the present study.

### Ethics of research using animals and clinical samples

All animal experiments using BALB/c nu/nu (9-week-old, female, Japan SLC Inc.), BALB/c (10-week-old, male and female, Japan SLC Inc.) and C.B-17/Icr-scid/scid Jcl (9-week-old, female, CLEA Japan, Inc.) were approved by the Animal Research Committee of Kyoto University, and performed according to the guidelines governing animal care in Japan. The study protocols using human breast and lung cancer samples were approved by the Ethics Committee of Kyoto University Hospital. Informed consent was obtained from all patients. The study was performed in accordance with the Helsinki Declaration. Preparations of tissue microarrays mounted with human lung tumours were described previously^17^,^18^.

### Statistical analyses

The significance of differences between two independent subjects and among multiple subjects was determined using the Student’s *t*-test and Dunnett’s test, respectively. The Kaplan–Meier method was used to evaluate patients’ prognoses and log-rank tests were used to compare survival rates among groups. A *P*-value <0.05 was considered to be significant.

## Author contributions

Y.G. performed the experiments, analysed the data and co-wrote the manuscript. L.Z., C.J.Y., Y.Z., K.H., S.I., M.Y., H.K. and E.M.H. contributed to the data analysis and discussion. A.M. and K.S. contributed to the data analysis and technical support. M.K. constructed the HIF-1 expression vector. K.T. contributed to the immunoprecipitation data analysis and discussion. M. Torii and M. Toi contributed to the research using human breast cancer samples. T.S., T.M., M.S. and H.D. contributed to the research using human lung cancer samples. M.H. supervised the project. H.H. designed and supervised the study, analysed the data and co-wrote the manuscript.

## Additional information

**How to cite this article:** Goto, Y. *et al.* UCHL1 provides diagnostic and antimetastatic strategies due to its deubiquitinating effect on HIF-1α. *Nat. Commun.* 6:6153 doi: 10.1038/ncomms7153 (2015).

## Supplementary Material

Supplementary Information Supplementary Figures 1-20.

## Figures and Tables

**Table 1:** The relationship between HIF-1**α** and UCHL1 expression levels in human breast cancers.

	**HIF-1α levels**		
	**Low (+)**	**Intermediate (++)**	**High (+++)**
*UCHL1 levels*			
Low (+)	4 (13%)	0 (0%)	0 (0%)
Intermediate (++)	2 (7%)	7 (23%)	5 (17%)
High (+++)	0 (0%)	3 (10%)	9 (30%)

The relationship between HIF-1α and UCHL1 expression levels was analysed in human breast cancers.

**Table 2:** The relationship between HIF-1**α** and UCHL1 expression levels in human lung cancers.

	**HIF-1α levels**	
	**Low (− and +)**	**High (++ and +++)**
*UCHL1 levels*		
Low (− and +)	94 (37%)	117 (46%)
High (++and +++)	3 (1%)	40 (16%)

The relationship between HIF-1α and UCHL1 expression levels was analysed in human lung cancers (*χ*^2^ test, *P*<0.0001).
