# Supplementary material for: UCHL1 provides diagnostic and antimetastatic strategies due to its deubiquitinating effect on HIF-1α
Source: Nat Commun. 2015 Jan 23;6:6153. doi: 10.1038/ncomms7153 (PMC4317501; doi:10.1038/ncomms7153)
Supplement: Supplementary Information — Supplementary Figures 1-20. [file ncomms7153-s1.pdf]

Supplementary Figure 1

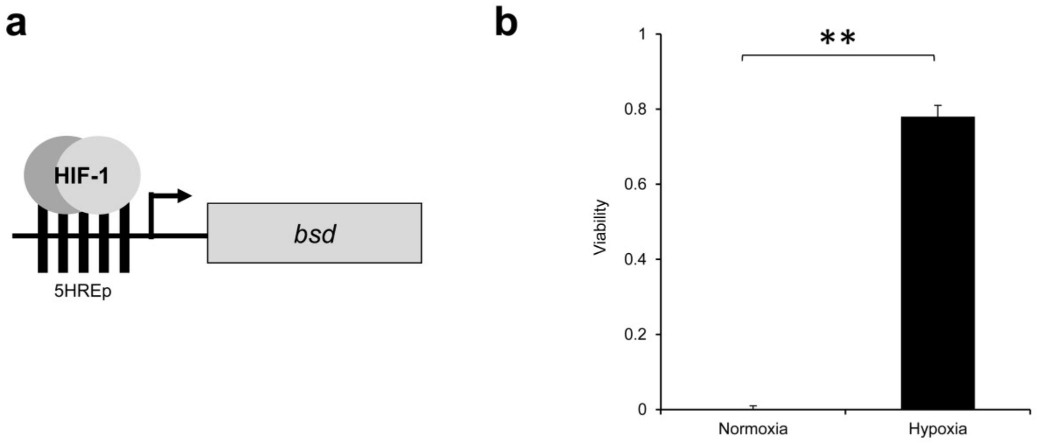

Supplementary Figure 1. Screening for novel genes responsible for the activation of HIF-1. (**a**) Schematic diagram of the p5HREp-bsd plasmid that expressed the blasticidin S-resistant gene (*bsd*) in a HIF-1-dependent manner. (**b**) NIH3T3/5HRE-BSD cells were cultured under normoxic (20%) and hypoxic (0.1%) conditions with 10 μM blasticidin S for 24 h. The fraction of surviving cells was quantified by Cell Count Reagent SF (Nacalai Tesque) according to the manufacturer's instructions. Mean ± s.d. n = 3, **p < 0.01 (Student's *t*-test between the indicated groups).

Supplementary Figure 2

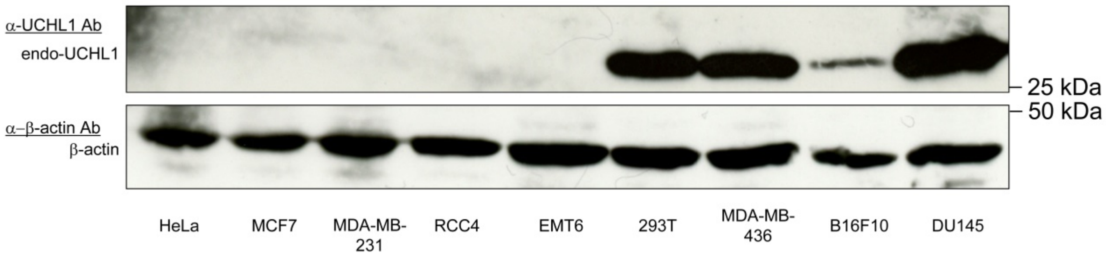

Supplementary Figure 2. Endogenous expression levels of UCHL1 in various cancer cell lines. Twenty-four hours after seeding the indicated cells, cell lysates were harvested and subjected to Western blotting with anti-UCHL1 (upper) and anti-β-actin (lower) antibodies.

Supplementary Figure 3

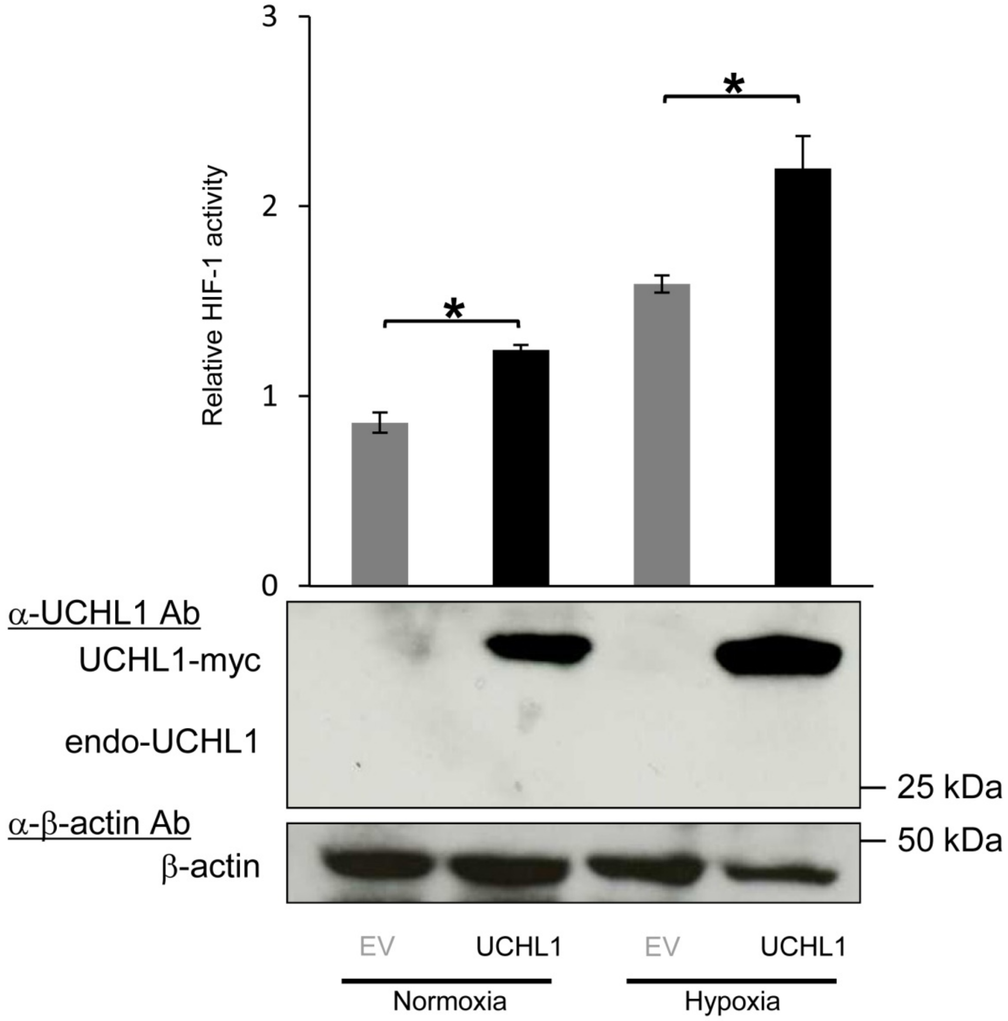

Supplementary Figure 3. Activation of HIF-1 activity by UCHL1 overexpression. MDA-MB-231/5HRE-Luc cells were transfected with the UCHL1-expression vector, pcDNA4/UCHL1 (UCHL1), or its empty vector, pcDNA4/myc-His A (EV), cultured under normoxic (20%) or hypoxic (1%) conditions for 24 h, and subjected to Western blotting using the indicated antibodies and the luciferase assay. Mean ± s.d. n = 3, *p < 0.05, **p < 0.01 (Student's *t*-test between the 2 indicated groups).

Supplementary Figure 4

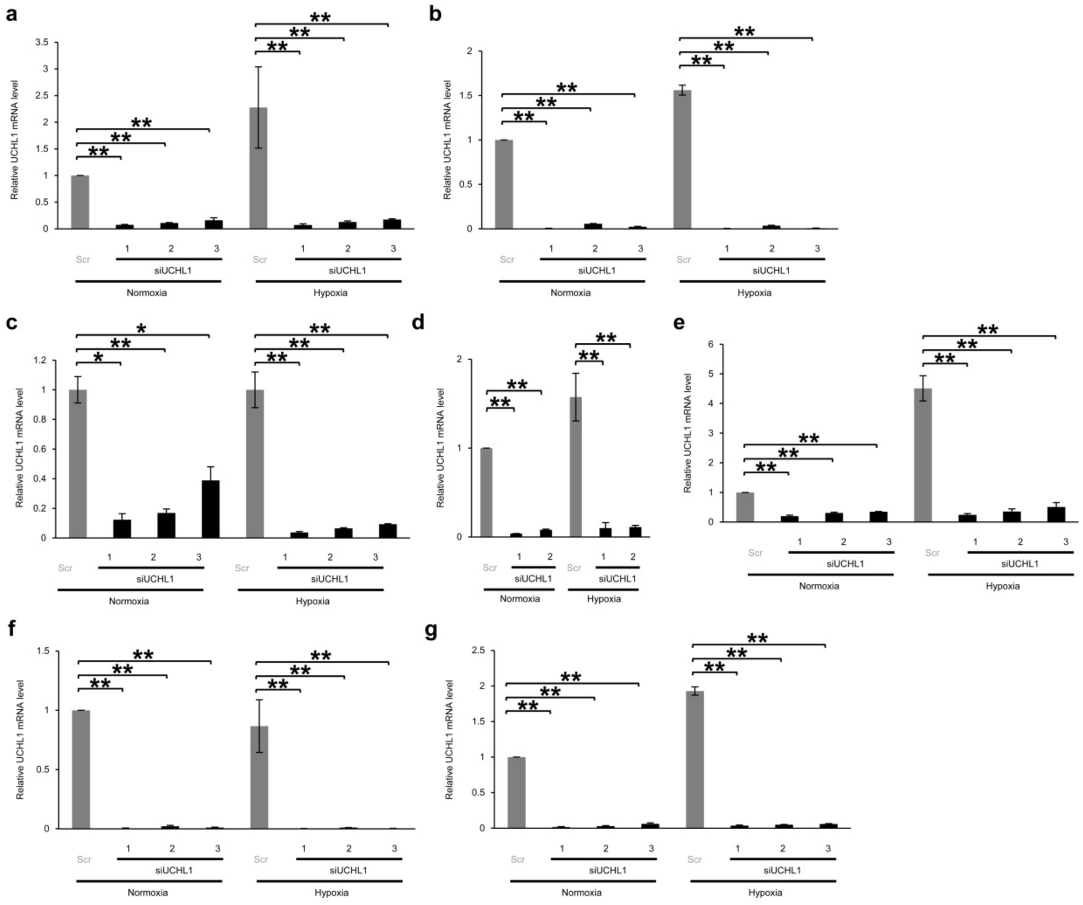

Supplementary Figure 4. Efficiency of UCHL1 knockdown. The efficiency of UCHL1 knockdown in HEK293T/5HRE-Luc cells in the experiment in Figure 1c (**a**), MDA-MB-436/5HRE-Luc cells in that of Figure 1c (**b**), HEK293T cells in that of Figure 1e and Supplementary Fig. 7 (**c**), HEK293T cells in that of Figure 2b (**d**), HEK293T cells in that of Figure 2d left (**e**), MDA-MB-436 cells in that of Figure 2d right (**f**), and DU145 cells in that of Supplementary Fig. 5 (**g**) were quantified by qRT-PCR. Mean ± s.d. n = 3, $*p < 0.05$, $**p < 0.01$ (Student's *t*-test between the 2 indicated groups).

Supplementary Figure 5

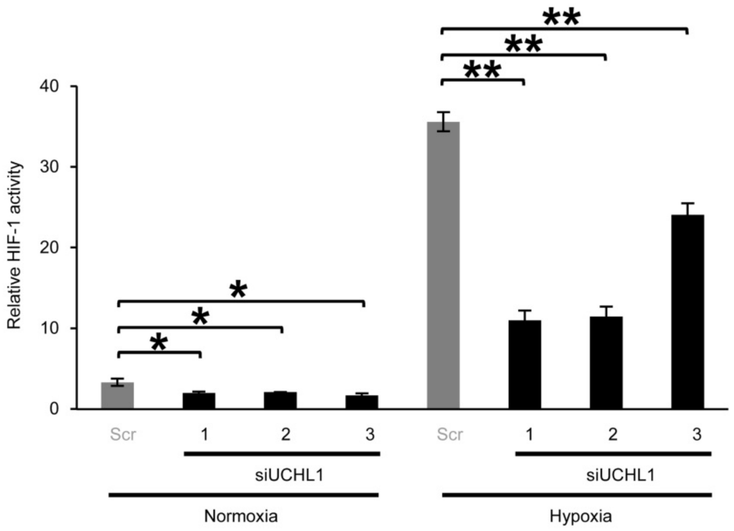

Supplementary Figure 5. Suppression of HIF-1 activity by UCHL1 knockdown. DU145 cells were transfected with a plasmid encoding the *5HREp-luc* plasmid for 24 h, and treated with scramble- (Scr) or UCHL1-siRNA (siUCHL1), cultured under normoxic (20%) or hypoxic (1%) conditions for 24 h, and then subjected to the luciferase assay. Mean ± s.d. n = 3, *p < 0.05, **p < 0.01 (Student's *t*-test between the 2 indicated groups).

Supplementary Figure 6

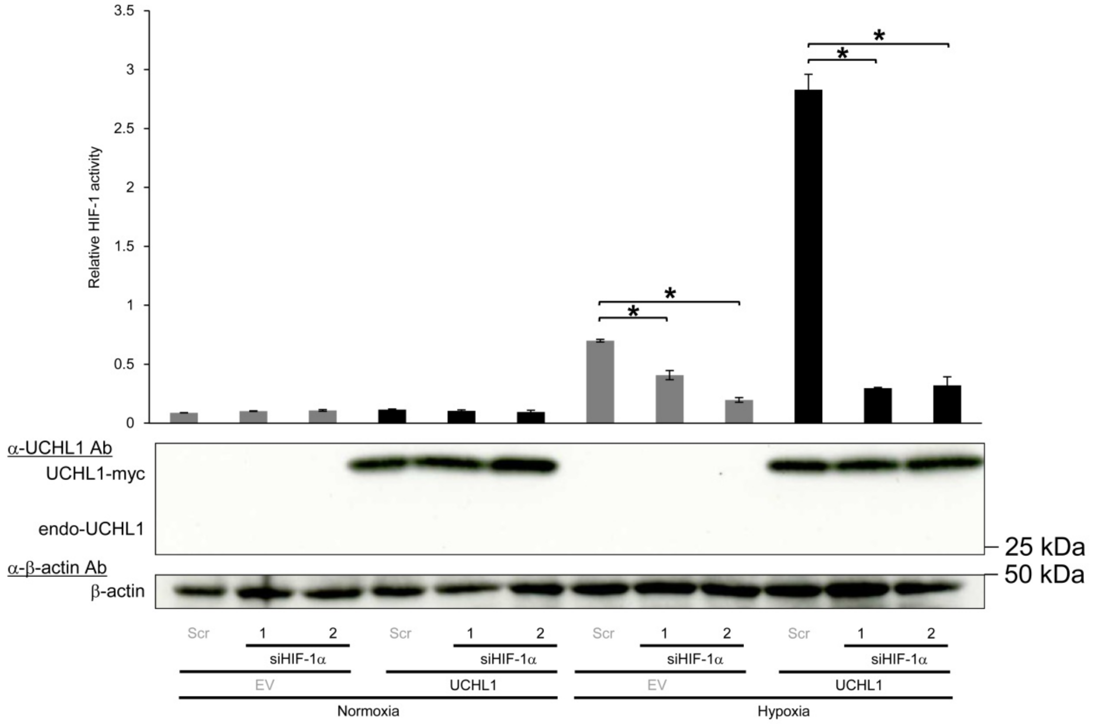

Supplementary Figure 6. Involvement of HIF-1α in UCHL1-dependent upregulation of *5HRE-luc* reporter activity. HeLa/5HRE-Luc cells were transfected with the UCHL1-expression vector, pcDNA4/UCHL1 (UCHL1) or its empty vector, pcDNA4/myc-His A (EV), treated with either scramble- (Scr) or HIF-1α-siRNA (siHIF-1α), cultured under normoxic (20%) or hypoxic (1%) conditions for 24 h, and then subjected to Western blotting using the indicated antibodies and luciferase assay. Mean ± s.d. n = 3, $*p < 0.05$ (Student's *t*-test between the 2 indicated groups).

Supplementary Figure 7

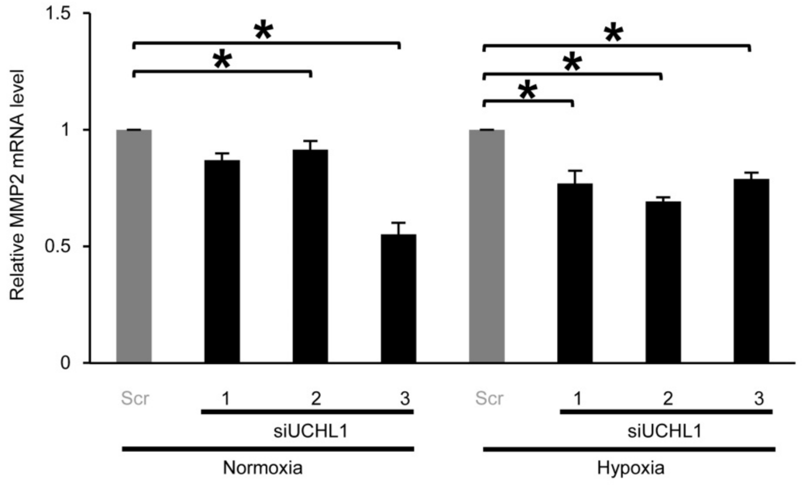

Supplementary Figure 7. Decrease in HIF-1-downstream gene expression by UCHL1 knockdown. 293T cells, which originally showed the high endogenous expression of UCHL1, were transfected with either scramble- (Scr) or UCHL1-siRNA (siUCHL1), cultured under normoxic (20%) or hypoxic (1%) conditions for 24 h, and then subjected to qRT-PCR to quantify *MMP2* mRNA levels. Mean ± s.d. n = 3, *$p < 0.05$ (Student's *t*-test between the 2 indicated groups).

Supplementary Figure 8

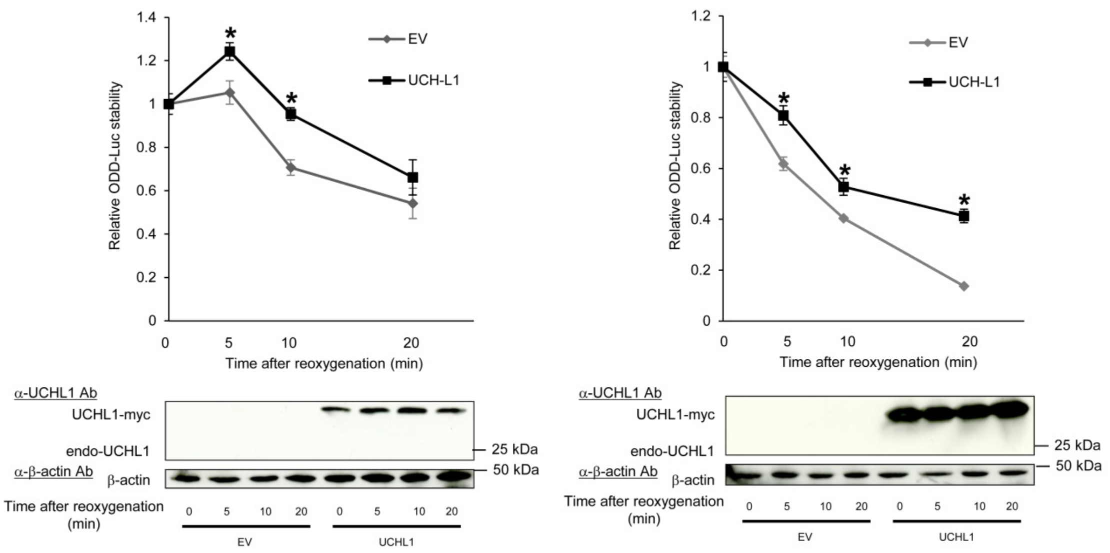

Supplementary Figure 8. Prolonged HIF-1α half-life by UCHL1 overexpression. HeLa/ODD-Luc (left) and MCF7/ODD-Luc (right) cells were transfected with the UCHL1-expression vector, pcDNA4/UCHL1 (UCHL1) or its empty vector, pcDNA4/myc-His A (EV), cultured under hypoxic conditions (1%) for 24 h, and treated with cycloheximide (10 μg/mL) under normoxic conditions (20%). After the reoxygenation treatment for the indicated period, cells were subjected to Western blotting using the indicated antibodies and luciferase assay. Mean ± s.d. n = 3, *$p < 0.05$ (Student's *t*-test between the 2 indicated groups) .

Supplementary Figure 9

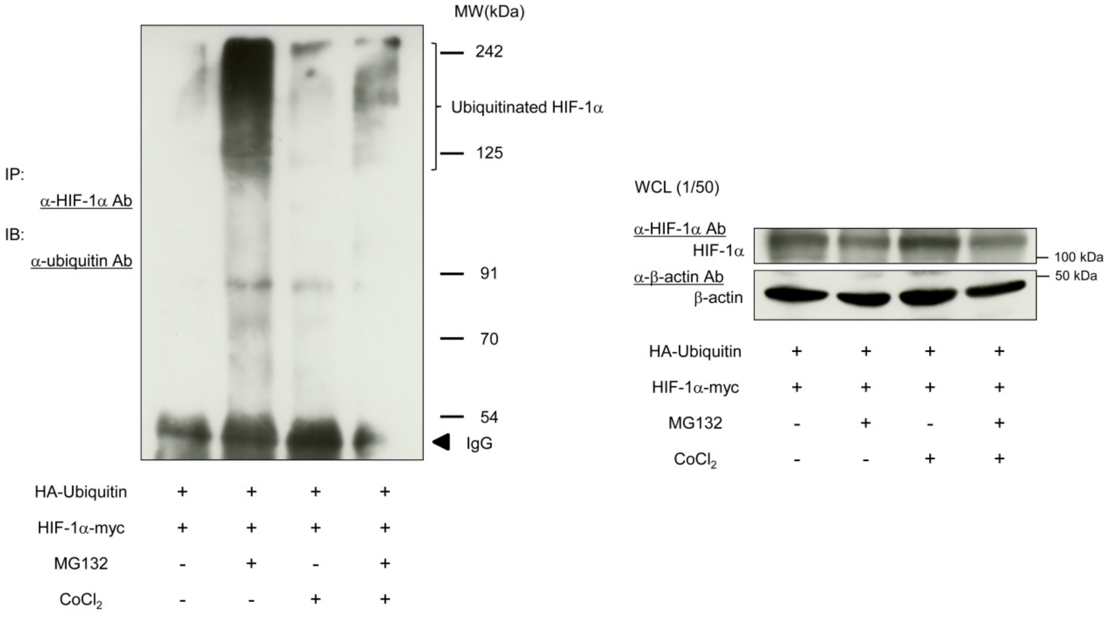

Supplementary Figure 9. Quantitative analysis of the ubiquitination of HIF-1α. 293T cells, which originally showed the high endogenous expression of UCHL1, were transiently transfected with the expression vector for HIF-1α, pcDNA4A/HIF-1α-myc, and the expression vector for HA-tagged ubiquitin, pMT132, and were then treated with the indicated combination of the proteasome inhibitor, MG132 (30 μM), and PHD inhibitor, $CoCl_2$ (800 μg/mL), under normoxic conditions (20%) for 24 h. Cell lysates were used for immunoprecipitation with the anti-HIF-1α antibody and subjected to Western blotting with the anti-ubiquitin antibody. One fiftieth of the whole cell lysate (WCL) was subjected to Western blotting with the indicated antibodies.

Supplementary Figure 10

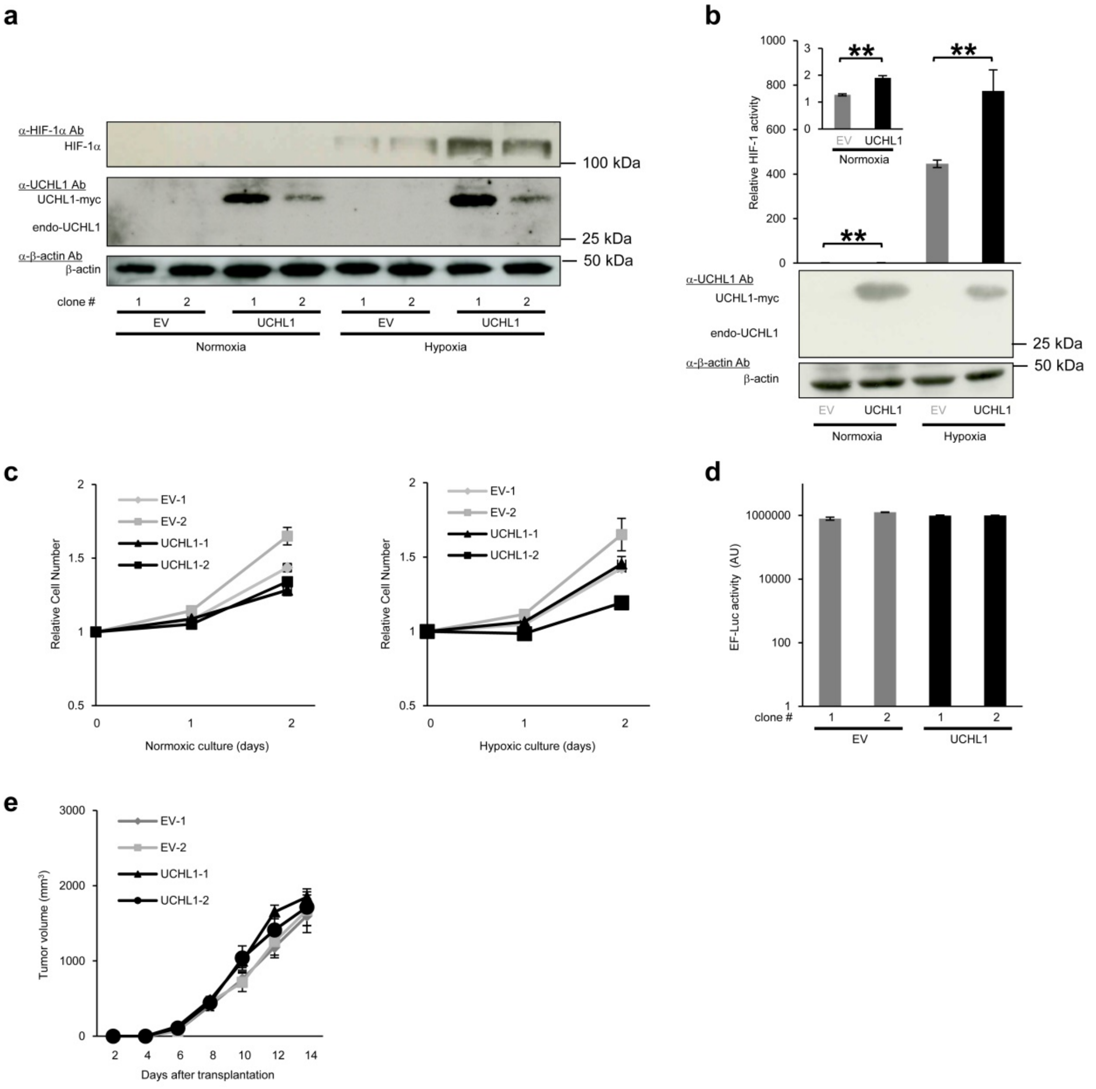

Supplementary Figure 10. Characterization of EMT6/EF-Luc/UCHL1 and EMT6/EF-Luc/EV stable transfectants. (**a**) EMT6/EF-Luc/EV-1, 2 and EMT6/EF-Luc/UCHL1-1, 2 cells were cultured under normoxic (20%) and hypoxic (0.1%) conditions for 24 h, and then subjected to Western blotting with the indicated antibodies. (**b**) EMT6/5HRE-Luc cells were transfected with the UCHL1-expression vector, pcDNA4/UCHL1 (UCHL1) or its empty vector, pcDNA4/myc-His A (EV), cultured under normoxic (20%) or hypoxic (1%) conditions for 24 h, and then subjected to Western blotting using the indicated

antibodies and luciferase assay. Mean ± s.d. **$p < 0.01$. (Student's *t*-test between the 2 indicated groups) (**c**) EMT6/EF-Luc/EV-1, 2 and EMT6/EF-Luc/UCHL1-1, 2 cells were cultured under normoxic (20%) and hypoxic (1%) conditions for the indicated period. The growth of cells was monitored with Cell Count Reagent SF (Nacalai Tesque) according to the manufacturer's instructions. (**d**) Twenty-four hours after seeding the stable transfectants, EMT6/EF-Luc/EV-1, 2, and EMT6/EF-Luc/UCHL1-1, 2 cells, cells were subjected to the luciferase assay. n = 3, Mean ±s.d. (**e**) The growth of the tumor xenografts was monitored after the subcutaneous transplantation of the EMT6/EF-Luc/EV-1, 2 and EMT6/EF-Luc/UCHL1-1, 2 cell suspensions (3×10^5 cell/mouse) into the right legs of athymic nude mice. The tumor volume was calculated as $0.5 \times \text{length} \times \text{width}^2$. n = 6.

Supplementary Figure 11

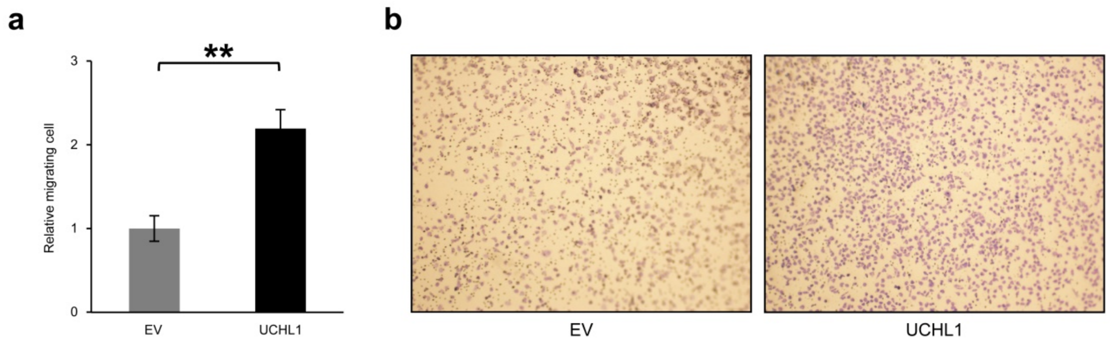

Supplementary Figure 11. Transwell migration assay with or without UCHL1 overexpression. The transwell migration assay using EMT6/EF-Luc/EV (EV) and EMT6/EF-Luc/ /UCHL1 (UCHL1) cells. Mean ± s.d. n = 4, *p < 0.05 (Student's *t*-test between the 2 indicated groups). Representative images are shown in **b**.

Supplementary Figure 12

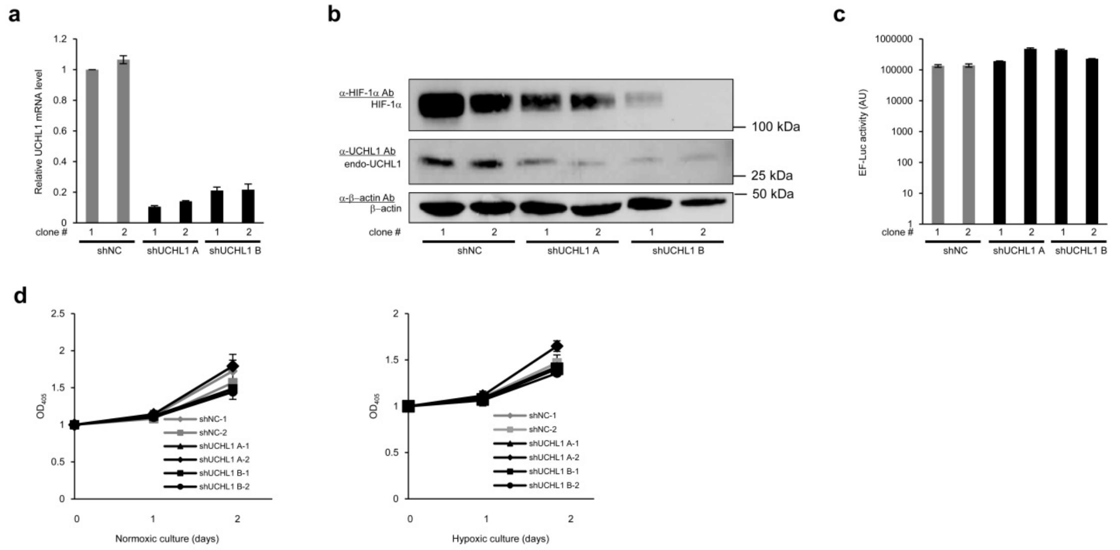

Supplementary Figure 12. Characterization of B16F10/EF-Luc/shUCHL1 and B16F10/EF-Luc/shNC stable transfectants. (**a**) B16F10/EF-Luc/shNC-1, 2 and B16F10/EF-Luc/shUCHL1 A-1, 2, B-1, and 2 cells were cultured under normoxic (20%) conditions for 24 h, and then subjected to qRT-PCR to quantify UCHL1 mRNA levels. Mean ± s.d. n = 3. (Student's *t*-test between the 2 indicated groups) (**b**) B16F10/EF-Luc/shNC-1, 2 and B16F10/EF-Luc/shUCHL1 A-1, 2, B-1, and 2 cells were cultured under hypoxic conditions (0.1%) for 24 h, and then subjected to Western blotting with the indicated antibodies. (**c**) Twenty-four hours after seeding stable transfectants, cells were subjected to the luciferase assay. (**d**) B16F10/EF-Luc/shNC-1, 2 and B16F10/EF-Luc/shUCHL1 A-1, 2, B-1, 2 cells were cultured under normoxic (20%) and hypoxic (1%) conditions for the indicated period. The growth of these cells was monitored with Cell Count Reagent SF (Nacalai Tesque) according to the manufacturer's instructions. n = 3, Mean ±s.d.

Supplementary Figure 13

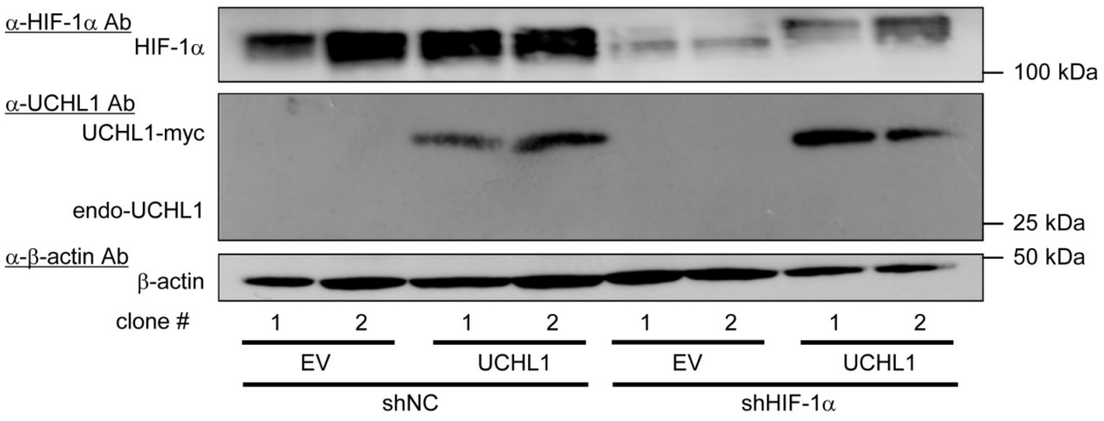

Supplementary Figure 13. Characterization of EMT6/EF-Luc/shNC/EV, EMT6/EF-Luc/shNC/UCHL1, EMT6/EF-Luc/shHIF-1α/EV-, and EMT6/EF-Luc/shHIF-1α/UCHL1 stable transfectants.

EMT6/EF-Luc/shNC/EV (clone #1, 2), EMT6/EF-Luc/shNC/UCHL1 (clone #1, 2), EMT6/EF-Luc/shHIF-1α/EV (clone #1, 2), and EMT6/EF-Luc/shHIF-1α/UCHL1 (clone #1, 2) cells were cultured under hypoxic conditions (0.1%) for 24 h, and subjected to Western blotting with the indicated antibodies.

Supplementary Figure 14

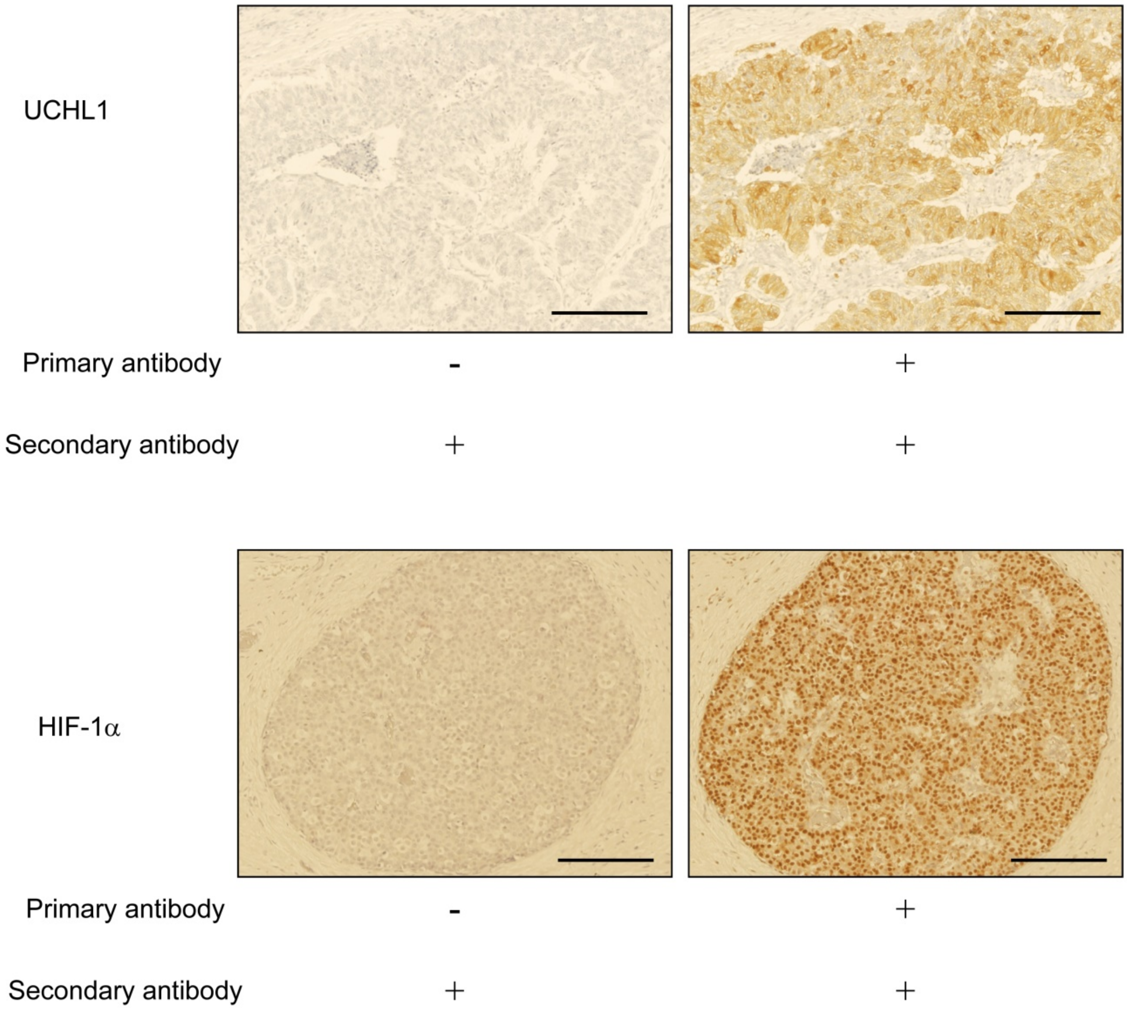

Supplementary Figure 14. Specificity of antibodies for immunohistochemical staining. Formalin-fixed paraffin-embedded sections of human breast tumors were subjected to immunohistochemical staining with (+) or without (-) anti-UCHL1 (upper) and anti-HIF-1α (lower) antibodies.

Supplementary Figure 15

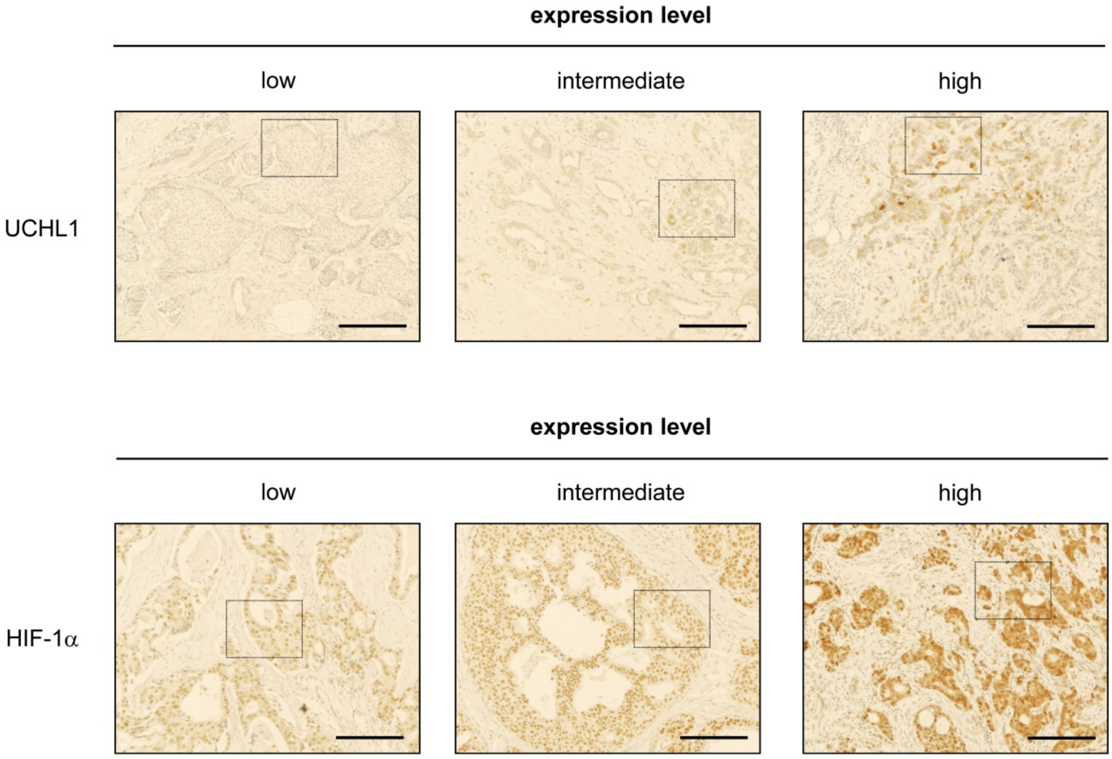

Supplementary Figure 15. Immunohistochemical analyses of UCHL1 and HIF-1α expression levels in human breast tumor sections. Low magnification images of Figure 6a and 6b (Immunohistochemical analyses using an anti-UCHL1 antibody [upper] and anti-HIF-1α antibody [lower]). Bar, 200 μm. Dotted squares represent the areas demonstrated in Figure 6a and 6b.

Supplementary Figure 16

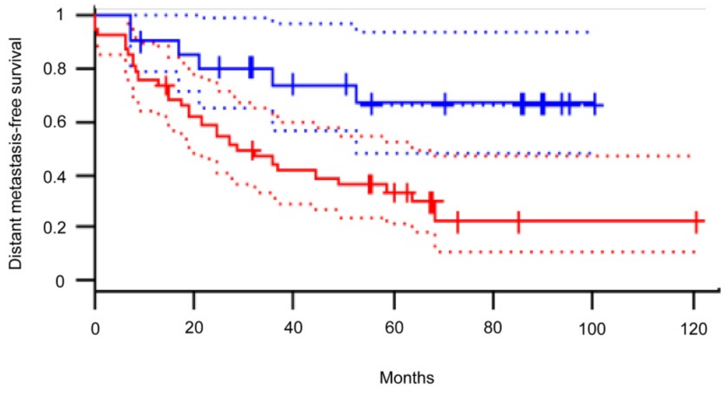

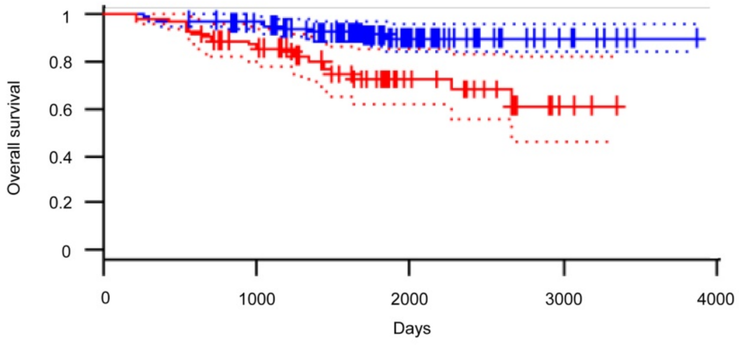

Supplementary Figure 16. Kaplan-Meier analysis of distant metastasis-free survival and overall survival rates of melanoma and lung adenocarcinoma patients. (**a**) **A** PrognoScan database-based Kaplan-Meier analysis of the distant metastasis-free survival rates of 63 melanoma patients stratified by high (red) and low (blue) UCHL1 levels (high: n = 42, low: n = 21). (**b**) **A** PrognoScan database-based Kaplan-Meier analysis of the overall survival rates of 204 lung adenocarcinoma patients stratified by high (red) and low (blue) UCHL1 levels (high: n = 73, low: n = 131).

Supplementary Figure 17

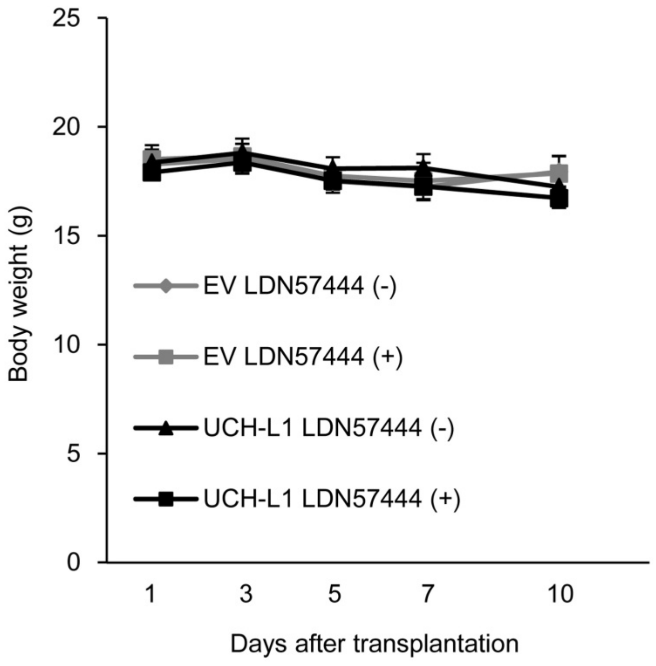

Supplementary Figure 17. No apparent side-effects due to the UCHL1 inhibitor. Changes in the body weights of EMT6/EF-Luc/EV- and EMT6/EF-Luc/UCHL1-tumor-bearing mice on the indicated days after the administration of DMSO and LDN57444. Mean ±s.d. n = 6.

Supplementary Figure 18

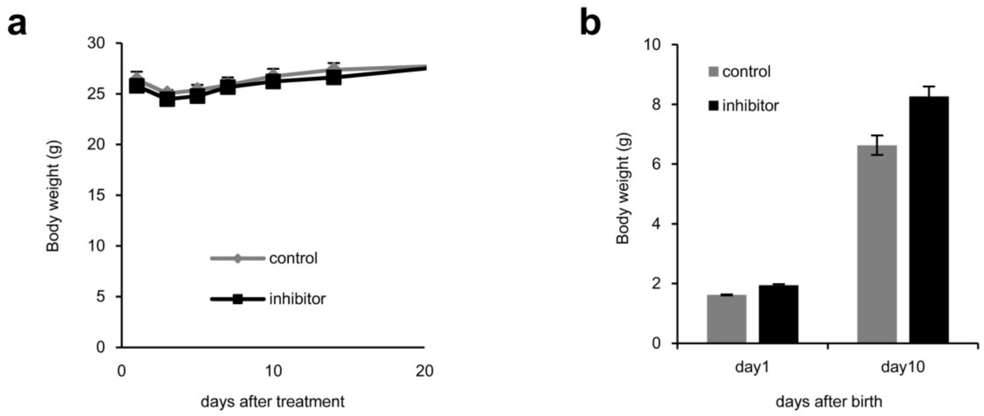

Supplementary Figure 18. No apparent side effects due to the UCHL1 inhibitor. (**a**) Changes in the body weights of 10-week-old BALB/c mice after the administration of DMSO or LDN57444 (0.5 mg/kg i.p.) on days 1, 3, and 5. Mean ± s.d. n = 4. (**b**) Ten-week-old male and female mice (4 pairs of BALB/c) were crossed for one week with the i.p. administration of DMSO or LDN57444 (0.5 mg/kg i.p.) on days 1, 3, and 5. The body weights of the resultant neonatal mice were measured 1 and 10 days after birth.

Supplementary Figure 19

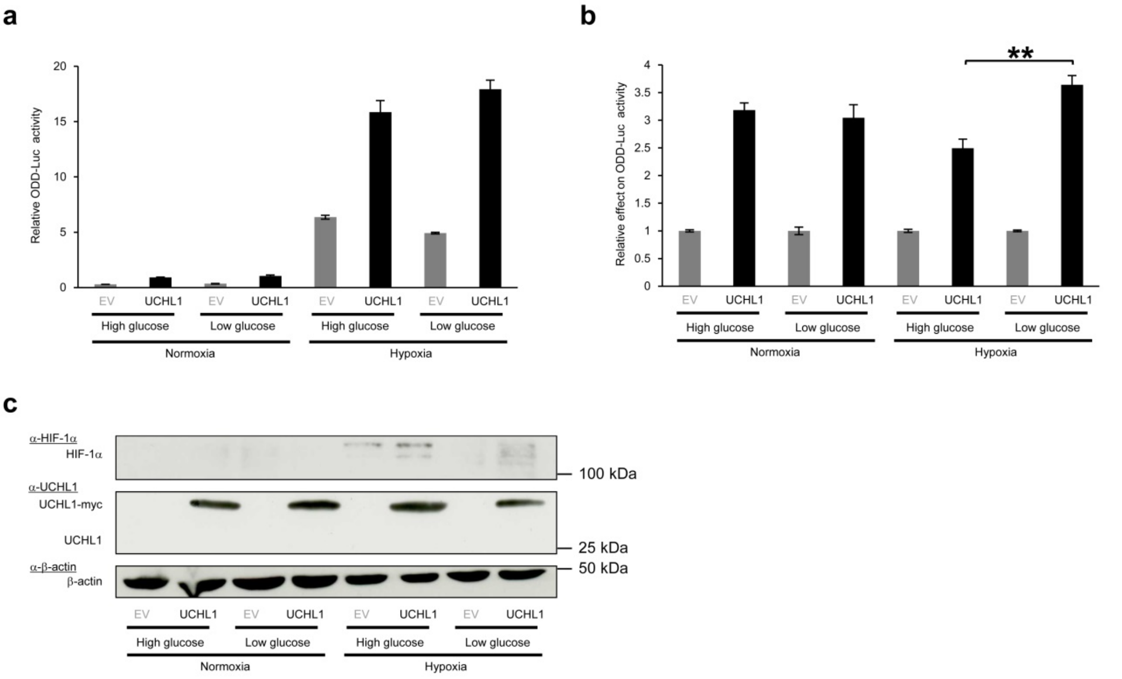

Supplementary Figure 19. UCHL1 efficiently stabilized the ODD-fusion protein under low glucose and hypoxic conditions. HeLa/ODD-Luc cells were transfected with either the UCHL1-expression vector, pcDNA4/UCHL1 (UCHL1), or its empty vector, pcDNA4/myc-His A (EV), and cultured in culture medium containing a low (0.45 g/L) or high (4.5 g/L) concentration of glucose under normoxic or hypoxic conditions for 24 h. (**a,b**) Cells were subjected to luciferase assay. Relative ODD-Luc activity in **a** was calculated as a ratio of *firefly* luciferase activity from the ODD-Luc expression vector to *renilla* luciferase activity from the internal control pRL-SV40. Relative effect on ODD-Luc activity in **b** was calculated as a ratio of the relative activity with UCHL1 transfection to that with EV transfection under each culture condition. Mean ± s.d. n = 3, *p < 0.01, NS = not significant (Student's *t*-test between the 2 indicated groups) . (**c**) Cells were then subjected to Western blotting for the indicated proteins.

Supplementary Figure 20

Fig. 1a left (α-UCHL1 Ab)

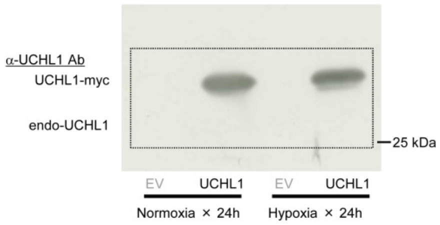

Fig. 1a left (α-β-actin Ab)

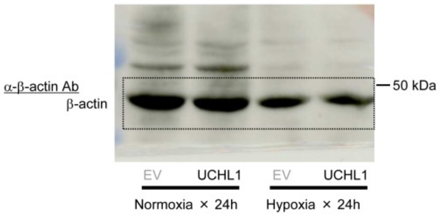

Fig. 1a right (α-UCHL1 Ab)

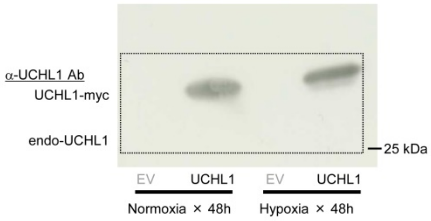

Fig. 1a right (α-β-actin Ab)

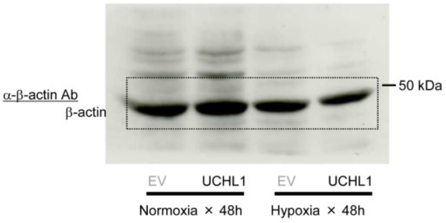

Fig. 1b left (α-UCHL1 Ab)

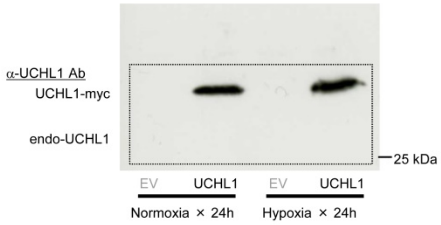

Fig. 1b left (α-β-actin Ab)

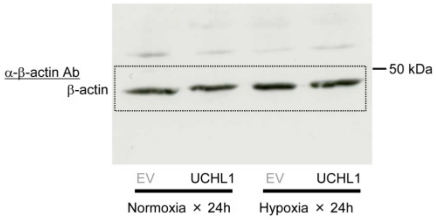

Fig. 1b left (α-UCHL1 Ab)

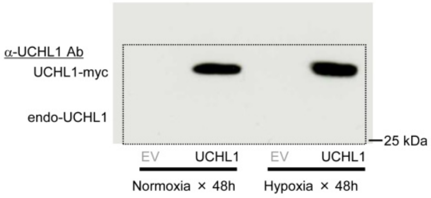

Fig. 1b left (α-β-actin Ab)

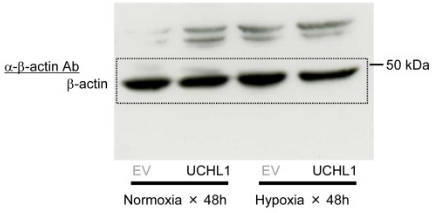

Fig. 1d (α-UCHL1 Ab)

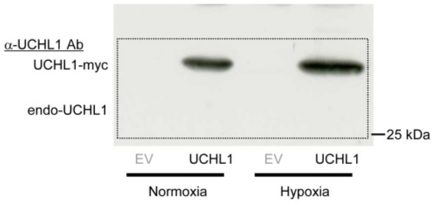

Fig. 1d(α-β-actin Ab)

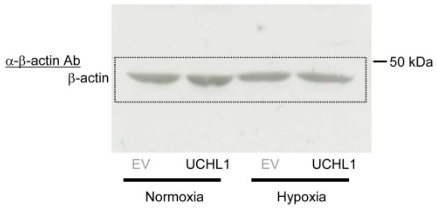

Supplementary Figure 20. Full blots of the indicated figures.

Fig. 2a (α-HIF1α Ab)

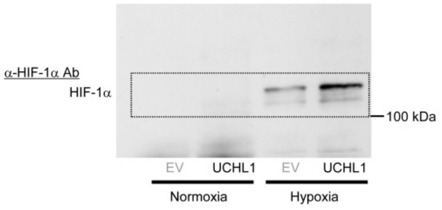

Fig. 2a (α-UCHL1 Ab)

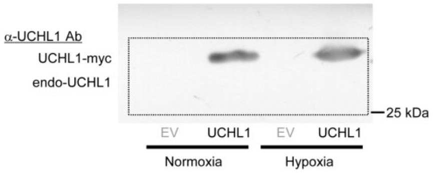

Fig. 2a(α-β-actin Ab)

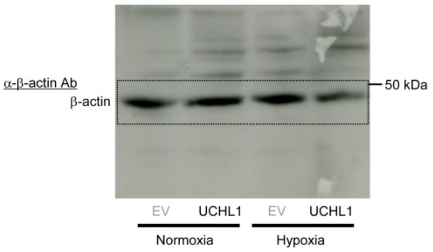

Fig. 2b (α-HIF1α Ab)

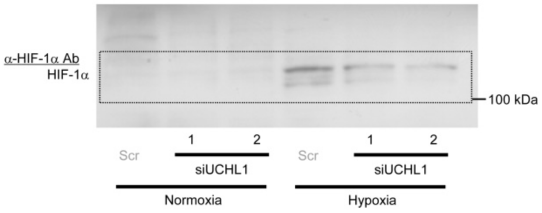

Fig. 2b(α-β-actin Ab)

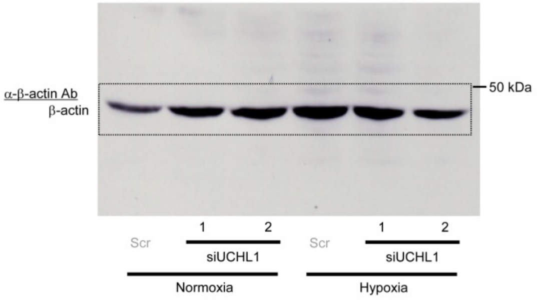

Fig. 2c left (α-UCHL1 Ab)

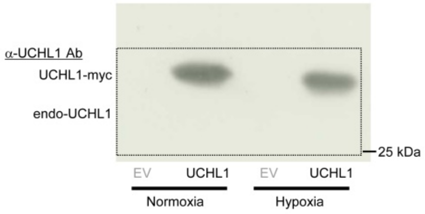

Fig. 2c left (α-β-actin Ab)

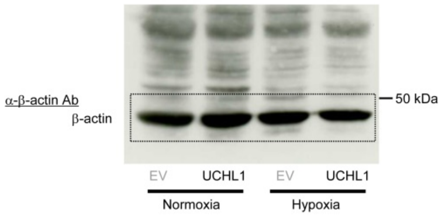

Fig. 2c right (α-UCHL1 Ab)

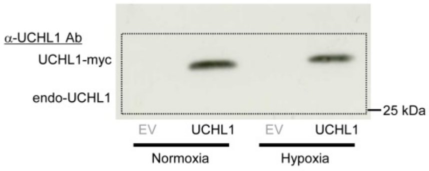

Fig. 2c right (α-β-actin Ab)

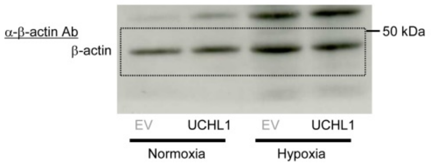

Fig. 2e (α-HIF1α Ab)

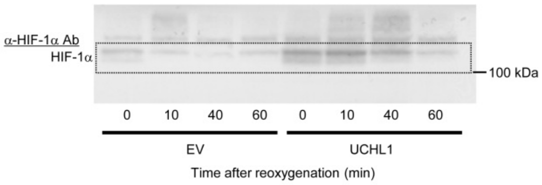

Supplementary Figure 20 *continued*.

Fig. 2e (α-UCHL1 Ab)

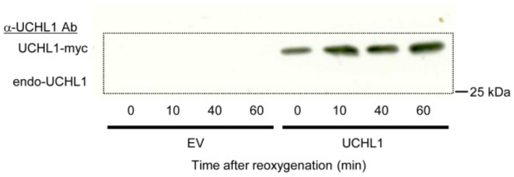

Fig. 2e (α-β-actin Ab)

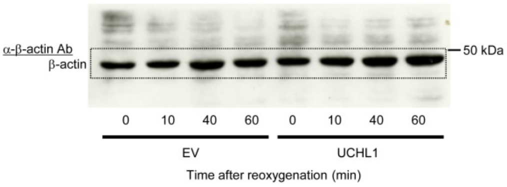

Fig. 2f (α-HIF1α Ab)

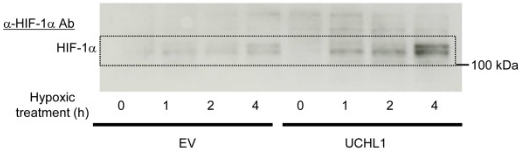

Fig. 2f (α-UCHL1 Ab)

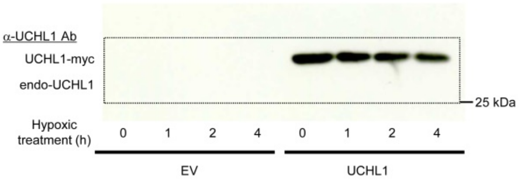

Fig. 2f (α-β-actin Ab)

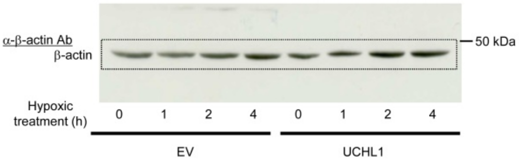

Fig. 3a (α-UCHL1 Ab)

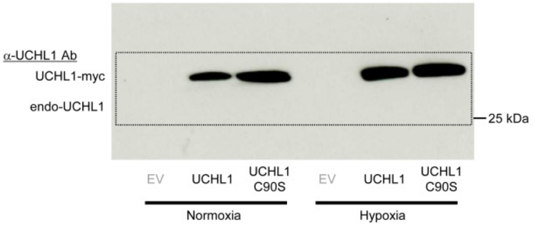

Fig. 3a (α-β-actin Ab)

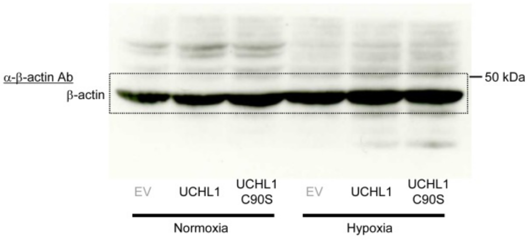

Fig. 3b (α-UCHL1 Ab)

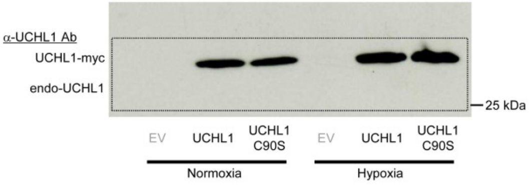

Fig. 3b (α-β-actin Ab)

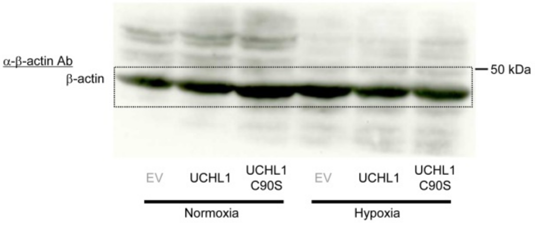

Supplementary Figure 20 *continued*.

Fig. 3c (α-Ubiquitin Ab)

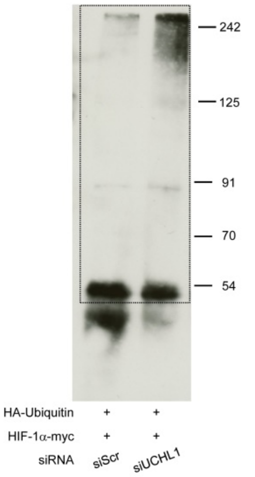

Fig. 3c (α-myc Ab)

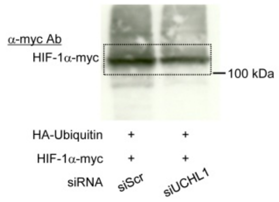

Fig. 3c (α-UCHL1 Ab)

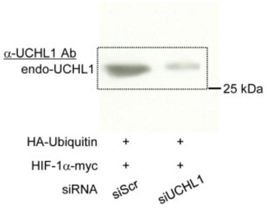

Fig. 3d (α-HIF1α Ab)

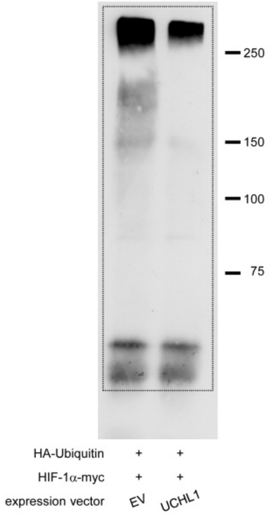

Fig. 3d (α-myc Ab)

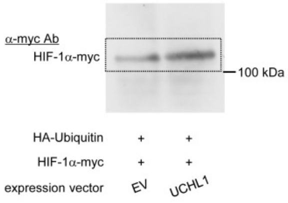

Fig. 3c (α-β-actin Ab)

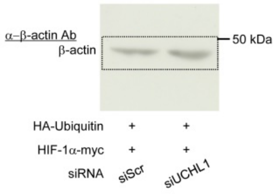

Fig. 3d (α-UCHL1 Ab)

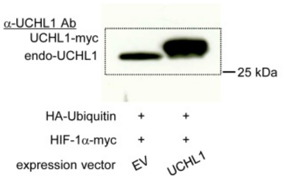

Fig. 3d (α-β-actin Ab)

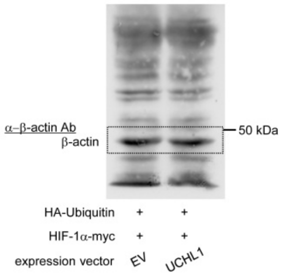

Fig. 3e α-UHIF-1α Ab

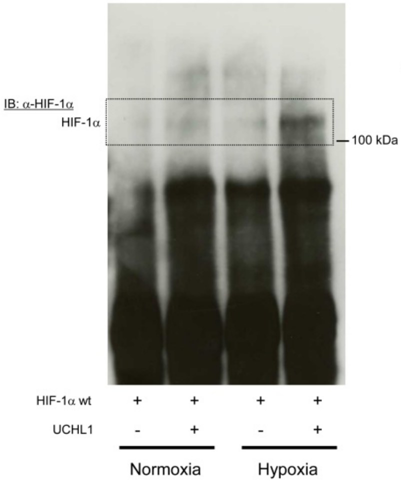

Fig. 3e α-UHIF-1α Ab

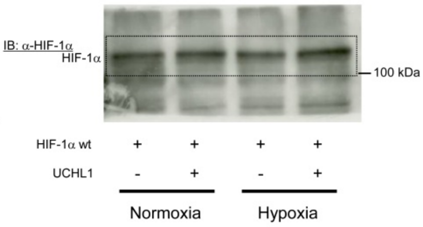

Supplementary Figure 20 ***continued*****.**

Fig. 3e α-UCHL1 Ab

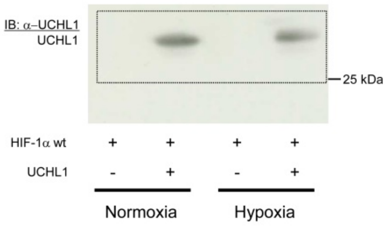

Fig. 3e α-β-actin Ab

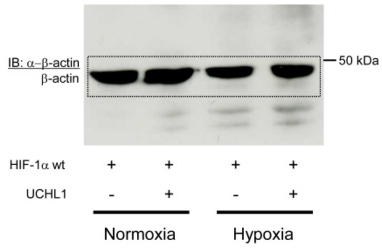

Fig. 3f (α-HIF1α Ab)

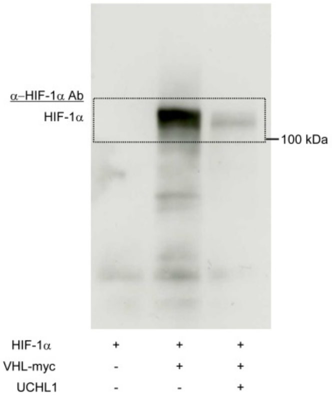

Fig. 3f (α-HIF1α Ab)

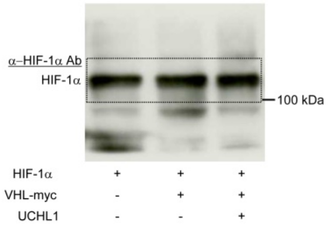

Fig. 3f (α-UCHL1 Ab)

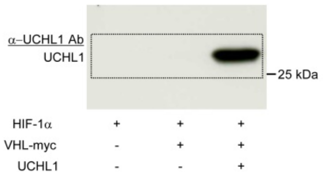

Fig. 3f (α-myc Ab)

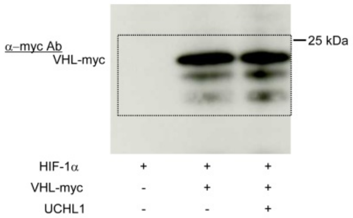

Fig. 3f (α-β-actin Ab)

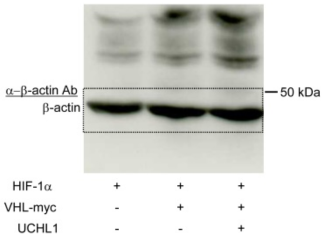

Fig. 3g left (α-UCHL1 Ab)

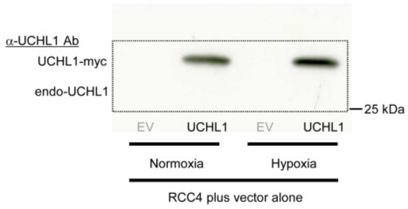

Fig. 3g left (α-β-actin Ab)

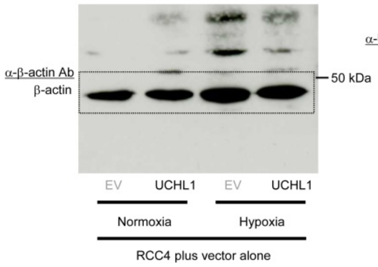

Fig. 3g right (α-UCHL1 Ab)

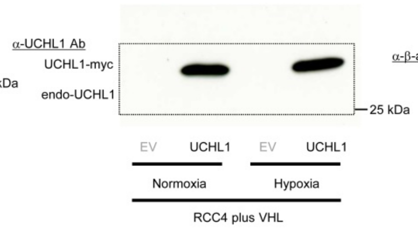

Fig. 3g right (α-β-actin Ab)

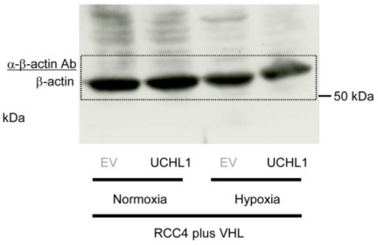

Supplementary Figure 20 *continued*.

Fig. 3h left (α-HIF1α Ab)

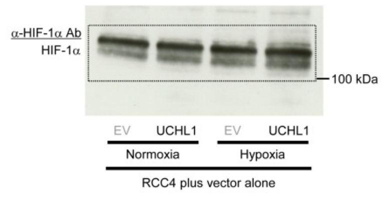

Fig. 3h left (α-UCHL1 Ab)

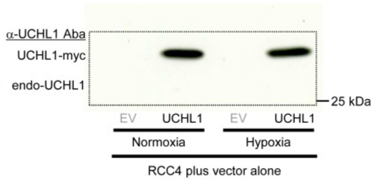

Fig. 3h left (α-β-actin Ab)

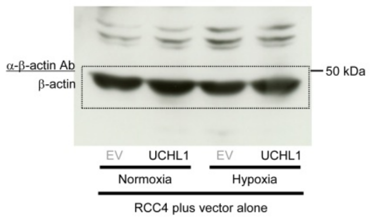

Fig. 3h right (α-HIF1α Ab)

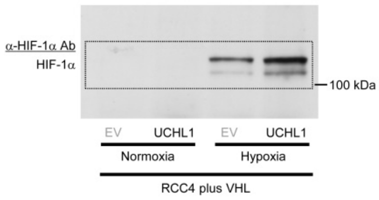

Fig. 3h right(α-UCHL1 Ab)

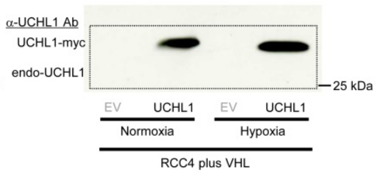

Fig. 3h right (α-β-actin Ab)

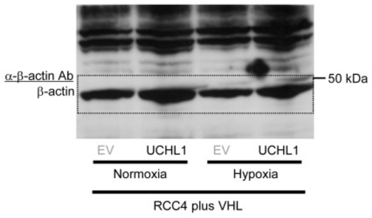

Fig. 4a (α-UCHL1 Ab)

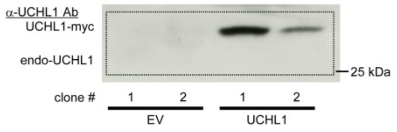

Fig. 4a (α-β-actin Ab)

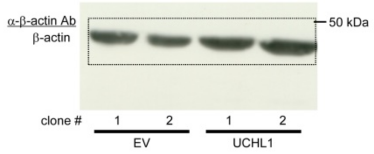

Fig. 4f (α-UCHL1 Ab)

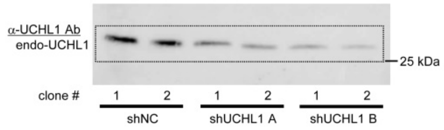

Fig. 4f (α-β-actin Ab)

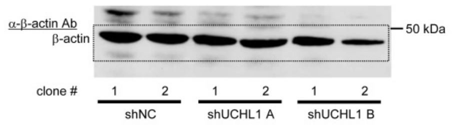

Fig. 7c (α-UCHL1 Ab)

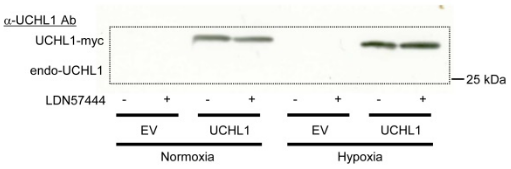

Fig. 7c (α-β-actin Ab)

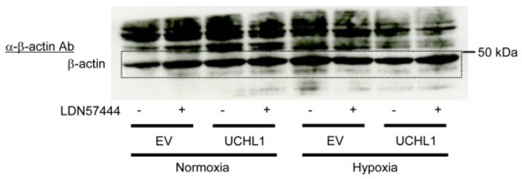

Supplementary Figure 20 *continued*.

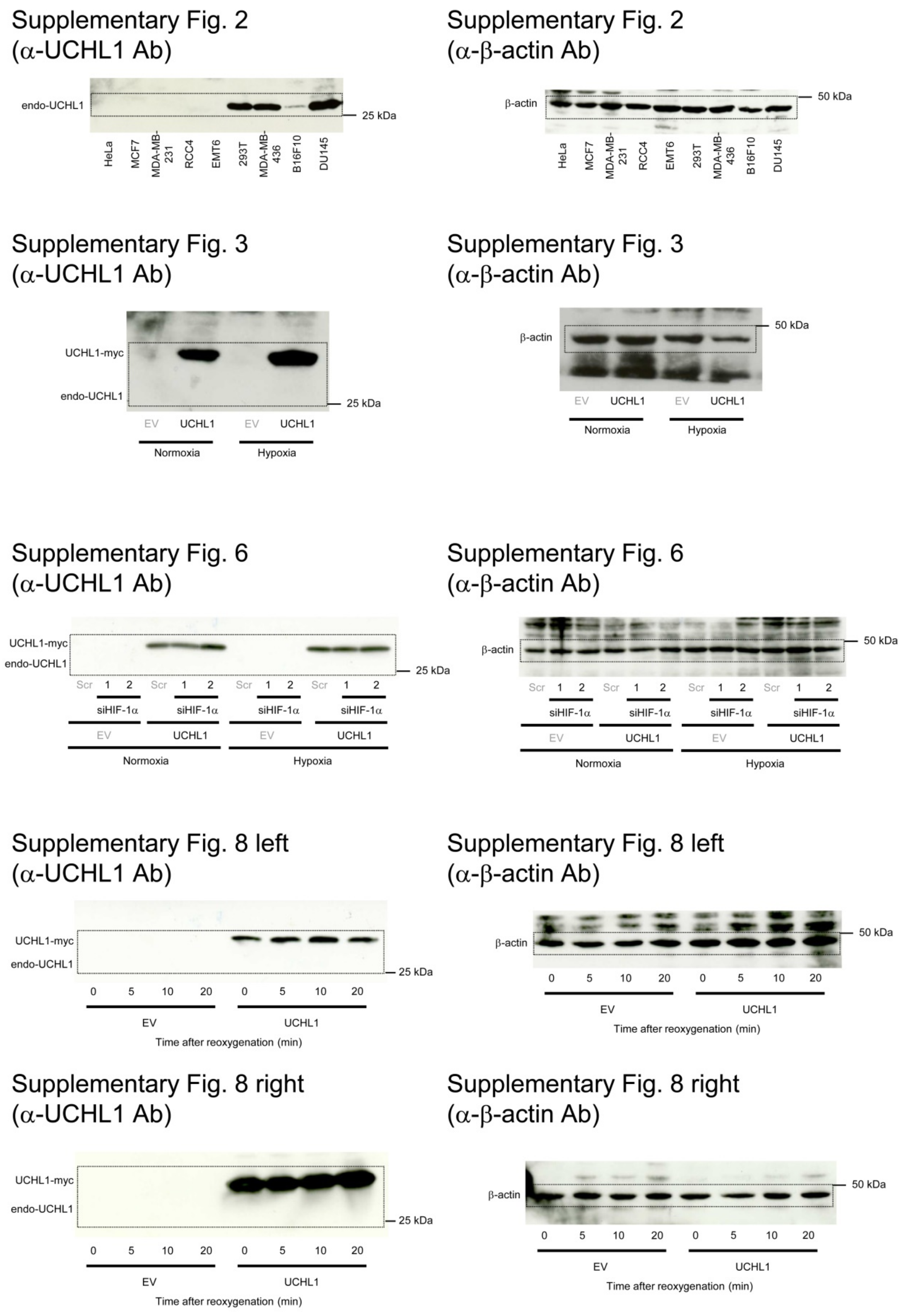

Supplementary Figure 20 *continued*.

Supplementary Fig. 9
(α-Ubiquitin Ab)

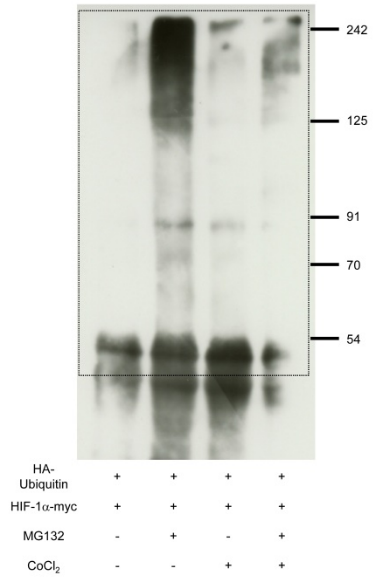

Supplementary Fig. 9
(α-HIF-1α Ab)

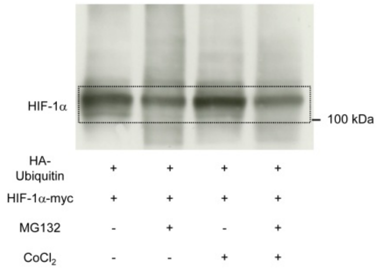

Supplementary Fig. 9
(α-β-actin Ab)

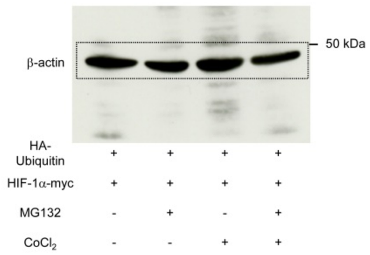

Supplementary Fig. 10a
(α-HIF1α Ab)

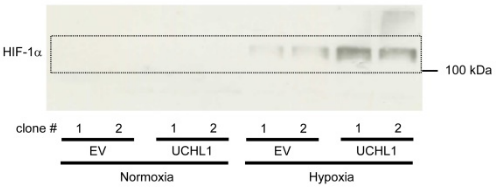

Supplementary Fig. 10a
(α-UCHL1 Ab)

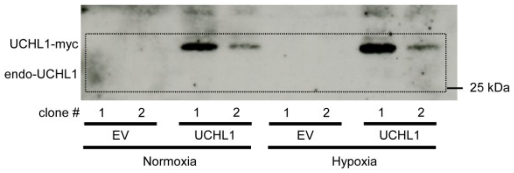

Supplementary Fig. 10a
(α-β-actin Ab)

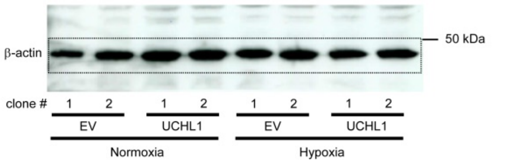

Supplementary Fig. 10b
(α-UCHL1 Ab)

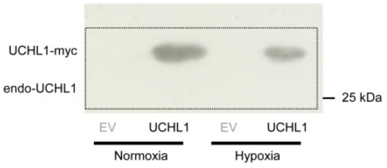

Supplementary Fig. 10b
(α-β-actin Ab)

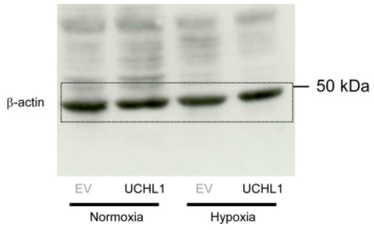

Supplementary Fig. 12b
(α-HIF1α Ab)

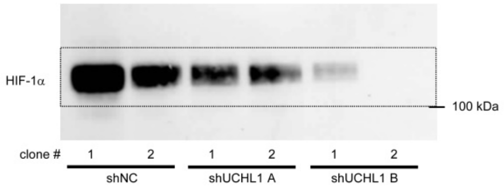

Supplementary Fig. 12b
(α-UCHL1 Ab)

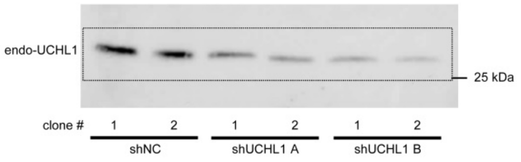

Supplementary Figure 20 *continued*.

Supplementary Fig. 12b
(α-β-actin Ab)

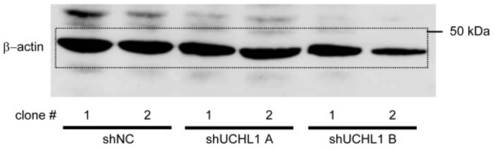

Supplementary Fig. 13
(α-HIF1α Ab)

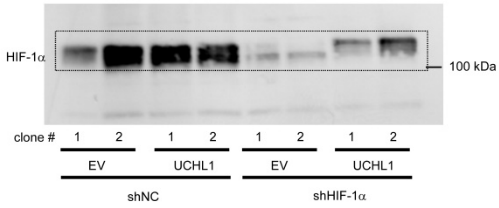

Supplementary Fig. 13
(α-UCHL1 Ab)

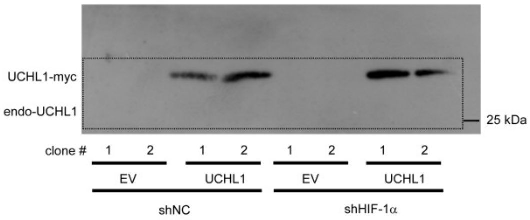

Supplementary Fig. 13
(α-β-actin Ab)

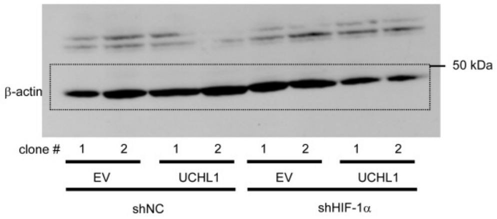

Supplementary Fig. 19b
. α-UHIF-1α Ab

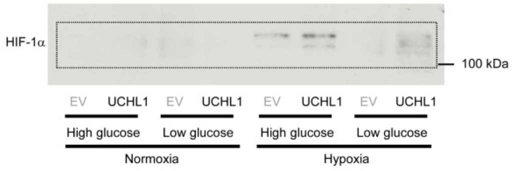

Supplementary Fig. 19b
α-UCHL1 Ab

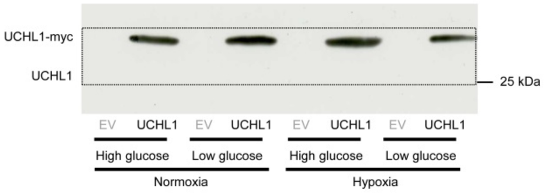

Supplementary Fig .19b
α-β-actin Ab

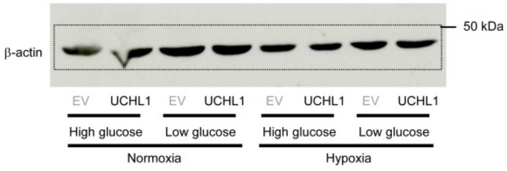

Supplementary Figure 20 ***continued.***
